# Patient-derived xenograft models: Current status, challenges, and innovations in cancer research

**DOI:** 10.1016/j.gendis.2025.101520

**Published:** 2025-01-08

**Authors:** Minqi Liu, Xiaoping Yang

**Affiliations:** Key Laboratory of Chemical Biology & Traditional Chinese Medicine Research of Ministry of Education, Key Laboratory of Study and Discovery of Small Targeted Molecules of Hunan Province, Engineering Research Center of Reproduction and Translational Medicine of Hunan Province, Key Laboratory of Protein Chemistry and Developmental Biology of Fish of Ministry of Education, Department of Pharmacy, School of Medicine, Hunan Normal University, Changsha, Hunan 410013, China

**Keywords:** Cancer models, Co-clinical trials, Drug screening, PDX models, Precision medicine

## Abstract

Despite advancing therapeutic treatments, cancer remains the leading cause of death worldwide, with most of its patients developing drug resistance and recurrence after initial treatment. Therefore, incorporating preclinical models that mimic human cancer biology and drug responses is essential for improving treatment efficacy and prognosis. Patient-derived xenograft (PDX) models, as a promising and reliable preclinical trial platform, retain key features of the original tumor such as gene expression profiles, histopathological features, drug responses, and molecular signatures more faithfully compared with traditional tumor cell line models and cell line-derived xenograft models. Their significant advantages have been the preferred choice in cancer research, especially demonstrating remarkable potential in drug development, clinical combination therapy, and precision medicine. However, the successful construction and effective application of PDX models still face several challenges. In this review, we summarize the details of constructing PDX models and the drivers affecting their success rates, which will provide some theoretical basis for subsequent model optimization. In the meantime, we delineate the strengths and weaknesses of various mature PDX models and other developing preclinical models, including PDX-derived models, organoids, and genetically engineered models. Moreover, we highlight the challenges of newly developed technologies on the PDX models. Finally, we emphasize the innovative usage of PDX models in a variety of cancer studies and offer insights into their prospects.

## Introduction

The incidence and prevalence of cancer have continued a sustained upward trend over the past several decades,^1^ with the number of newly diagnosed cancer patients projected to reach 28.4 million per year by 2040.^2^^,^^3^ Nowadays, most patients with advanced malignancies are still treated with conventional chemotherapy/radiotherapy and surgery, according to clinical guidelines, with minimal treatment options.^4^^,^^5^ Compared with traditional therapies, the arrival of therapeutic strategies such as precision medicine and immunotherapy has brought new hope for cancer treatment and significantly improved the survival rate of tumor patients. However, not all patients can experience the benefits of these new therapies, and some challenges remain, for example, the problem of drug resistance is almost inevitable.^5^

Therefore, it is of global significance to explore the developmental mechanisms of cancer and to improve diagnostic and treatment strategies. Among these, developing preclinical experimental models that precisely encapsulate tumor biology, genetic heterogeneity, and drug response has been a key to cancer research, and these models are increasingly becoming an important and indispensable tool for this basic research^6^^,^^7^. Patient-derived xenograft (PDX) models are xenografts formed by implanting patient-derived tumor tissue or cancer cells into immunodeficient mice^8^^,^^9^ and have become one of the most essential tools for bridging the gap between traditional animal models and clinical trials. This review summarizes the recent advances in PDX modeling in diverse cancer research areas and provides the prospects of PDX modeling, highlighting the challenges and innovations in this field.

## Methodology for modeling PDX

Historical use of animal tumor models dates back to the 1950s, with studies reporting the use of animal models in leukemia for drug discovery.^10^ Immediately thereafter, Toolan et al in 1951 succeeded in growing 33 cell line-derived xenograft tumor models by subcutaneously injecting human tumor cell suspensions into X-ray irradiated experimental animals.^11^ It was not until the advent of the nude mouse model in 1962 inaugurated a new era of PDX modeling,^12^ which was the first mouse strain capable of being used for PDX model construction, followed by the first successful implantation of tumor fragments from a patient’s colonic adenocarcinoma into nude mice by Rygaard and Povlsen in 1969.^13^ Since then, studies on PDX models have continued to evolve and progress, not only constructing numerous novel host models but also optimizing and innovating these models by combining several innovative technologies.

Numerous PDX biobanks have been globally established to collect diverse types of tumor tissues for facilitating preclinical testing of cancer therapies on PDX models as described in Table 1, allowing researchers to conveniently use these sources to match their research interests.

**Table 1:** International PDX biobanks.

Affiliation	Cancer type	Internet Link
National cancer institute (NCI) PDXNet	Pan-cancer	PDX (pdxnetwork.org)
NCI patient-derived models repository (PDMR)	Pan-cancer	Patient-Derived Models Repository (PDMR) (cancer.gov)
NCI Pediatric Preclinical In Vivo Testing Consortium (PIVOT)	Pediatric tumors	Pediatric Preclinical In Vivo Testing Consortium (PIVOT) – Advancing treatment options for children with cancer (preclinicalpivot.org)
EurOPDX consortium	Pan-cancer	Homepage - EuroPDX
PDX finder	Pan-cancer	CancerModels.Org - Find PDX, organoid, and cell line cancer models
Jackson laboratory	Pan-cancer	The Jackson Laboratory (jax.org)
Champions oncology	Pan-cancer	Champions Oncology
UHN princess margaret living biobank	Pan-cancer	Princess Margaret Living Biobank | UHN (uhnresearch.ca)
Center for patient derived models (CPDM)	Pan-cancer	Center for Patient-Derived Models | Dana-Farber Cancer Institute
Charles river laboratories	Pan-cancer	Patient-Derived Xenograft: PDX Models | Charles River (criver.com)
Washington university PDX development and trial center (WU-PDTC)	Pan-cancer	PDXdb: Washington University PDX Development and Trial Center | PDXdb (wustl.edu)
Crown bioscience	Pan-cancer	Preclinical Oncology CRO | Contract Research Drug Development Company (crownbio.com)
St. Jude Children’s research hospital, childhood solid tumor network (CSTN)	Childhood solid tumor	Childhood Solid Tumor Network | St. Jude Research (stjude.org)
ITCC-P4 platform	Pediatric tumors	ITCC P4 - Paediatric Preclinical Proof Of Concept Platform
Candiolo cancer institute	Gastric cancer and colorectal cancer	Home | Istituto di Candiolo - FPO - IRCCS
Luxembourg institute of health	Tumors of the nervous system	PRECISION-PDX » Luxembourg Institute of Health (lih.lu)
Vall d’Hebron institute of oncology	Breast carcinoma, pancreatic cancer, colorectal cancer	Inicio - VHIO

## Procedure of PDX model building

While PDX models are usually based on tumor tissue obtained during surgery or biopsy, some studies have reported that patient-derived ascites,^14^ circulating tumor cells,^15^ or pleural fluid-obtained tumor cells^16^ can also effectively construct PDX models in certain tumor types. Moreover, radical surgical resection had better modeling success than partial resection or biopsy,^17^^,^^18^ whereas clinical biopsy specimens exhibited higher implantation rates in constructing metastatic PDX models.^19^^,^^20^ Patient-derived tumors were implanted as tumor fragments or single-cell suspensions into suitable immunodeficient mice (“F0” for the first generation, the sequent generations were named F1, F2, F3, … and Fn accordingly).^21^ Selecting to implant as tumor fragments better preserves intercellular interactions and mimics the tumor microenvironment, with more fragments and smaller sizes resulting in higher tumor grafting success rates.^6^^,^^22^ In contrast, the single-cell suspension avoids heterogeneity within the tumor to some extent, allowing researchers to perform unbiased collection and implantation of tumor samples,^23^ However, the pre-treatment process may result in a reduction of cellular activity due to chemical or physical damages, affecting success rates.^6^

**Figure 1:**
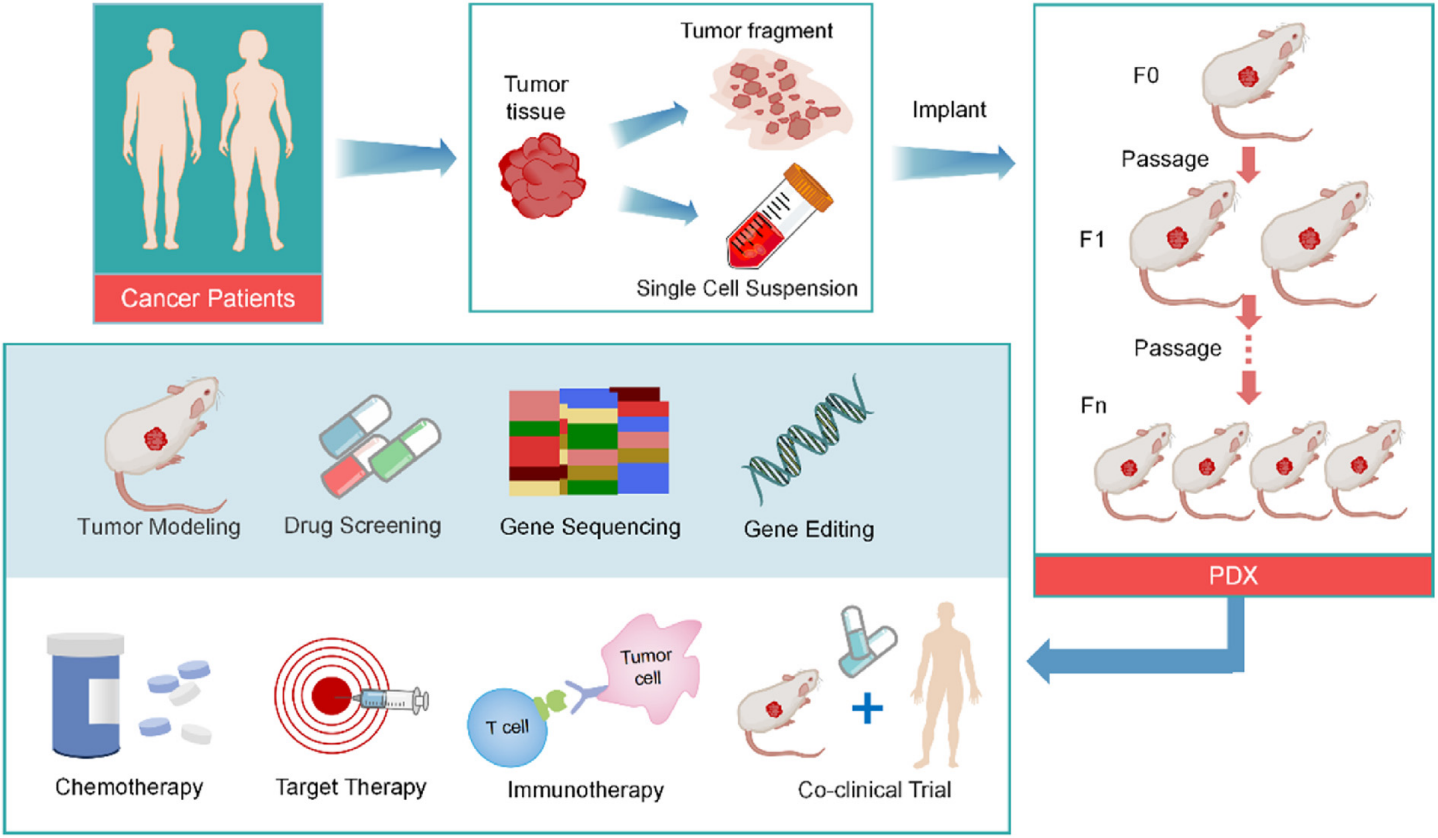
The steps of establishing PDX models. F0 refers to direct tumor tissue implantation into immunodeficient mice. Each subsequent successful transplantation generation is named F1, F2, … and Fn.

Tumor tissues or cells can be either mixed with basement membrane matrix (Matrigel) or directly embedded prior to transplantation, and the growth of the tumors mixed with Matrigel showed higher growth efficiency.^24^ Constructing the PDX model for different tumor types requires different times, thus, consistent tumor growth rate and volume monitoring are also essential parts of the modeling process.^25^^,^^26^ Usually, starting in the F3 generation, these mouse models were used for subsequent drug therapy trials, mechanistic studies, basic research, *etc*.^21^ (Fig. 1). Implantation failure can only be recognized when there is a significant trend of undetectable tumor growth for at least 6 months or the observed mass is only proliferated by non-epithelial cells.^27^^,^^28^ Moreover, PDX samples and data should be stored together with the patient’s clinical information to generate PDX libraries.^12^

## Interfering factors in the success rate of PDX modeling

Successful construction of PDX models undoubtedly presents innovative opportunities for oncology research. However, their success rate is influenced by various factors, which are discussed as follows.

## Immunodeficient mice

Mammals (especially mice and rats) have a higher similarity to humans than other non-mammals,^29^ and the mouse genome revealed 78.5% genetic homology between mice and humans.^30^^,^^31^ The mouse model also has relatively short generation cycles, quick reproduction rate, easy manipulation, and convenient allowance for genetic engineering and invasive experiments.^3^ To avoid immune rejection in mouse models, researchers choose mice with a high degree of immunodeficiency to create PDX models (Table 2).^3^

**Table 2:** Immunodeficient mouse species for PDX model construction.

Mice		Nude	SCID	NOD-SCID	NOG/NSG/NOJ	BRG/BRJ
Mutated gene		Foxn1	Prkdc	SCID	SCID, IL2Rγ, Jak3	IL2Rγ, Jak3, Rag-2
Immunological phenotype	T Cells	–	–	–	–	–
	B Cells	+	–	–	–	–
	Natural killer cells	+	+	↓	–	–
	Macrophages	+	+	↓	↓	+
	Dendritic cells	+	+	↓	↓	+
Success rate of PDX		Low	Low	Moderate	High	High
Advantages		Easy observation of subcutaneous tumor size; easily accessible	Better implantation than nude mice	Better implantation than nude mice	Outstanding transplant success rates; longer lifespan; suitable for establishing human mice	Outstanding implantation success; radiation resistant; suitable for establishing human mice
Disadvantages		Lower transplantation success rate; functional B cells and natural killer cells; T-cell leakage	Leakage of T and B cells; functional natural killer cells; radiosensitivity	Spontaneous lymphoma; short life expectancy; radiosensitivity	Need strict specific pathogen-free conditions; expensive	Expensive

Note: FOXN1, forkhead box N1; Prkdc, protein kinase, DNA-activated, catalytic subunit; SCID, severely combined immunodeficient; IL2Rγ, IL-2 receptor subunit gamma; Jak3, Janus kinase 3; Rag-2, recombination activating gene 2. NK: natural killer cells, Mϕ: macrophages, DCs: dendritic cells.

Nude mice are the first known immunodeficient mouse strain and are among the most used mouse strains for constructing PDX models.^32^ Afterward, Makino et al identified non-obese diabetic (NOD) mice in 1980,^33^ and Bosma et al first described severely combined immunodeficient (SCID) mice in 1983.^34^ Then, researchers established the NOD/SCID mice by crossing NOD and SCID mice.^12^ Based on NOD-SCID mice, researchers further developed new immunodeficient mouse models by introducing mutations in IL2Rγ (IL-2 receptor subunit gamma) or Jak3 (Janus kinase 3), including NOG (NOD/Shi-scid IL2Rγ null), NOJ (NOD/scid Jak3 null), or NSG (NOD/scid IL2Rγ null) mice.^35^, ^36^, ^37^ Moreover, BRG (BALB/c Rag-2 null/IL2Rγ null) and BRJ (BALB/c Rag-2 null/Jak3 null) mice were newly constructed with the background of BALB/c mice for IL2Rγ or Jak3, and Rag-2 (recombination activating gene 2) knockout mice.^38^^,^^39^

Different mouse strains present varying degrees of immunosuppression, and tumor implantation rates are higher in mouse strains with more severe immunodeficiency (*e.g.*, BRG/BRJ, NOG/NSG/NOJ).^40^^,^^41^ Meanwhile, distinct mouse strains are suitable for a variety of diverse types of cancer studies. For example, SCID mice show higher success rates in colorectal cancer and breast cancer cell implantation,^42^ while NSG mice have higher implantation rates for neuroblastoma.^43^ Furthermore, young mice (6–8 weeks) showed better hosts for xenografts versus old ones.^27^

## Implantation sites for PDX models and preservation of tumor specimens

In constructing PDX models, the transplantation site is a key factor affecting tumor growth and migration characteristics,^44^ including heterotopic transplantation and orthotopic transplantation.^21^ In general, orthotopic transplantation is considered the preferred transplantation site because it is closer to the original tumor in terms of anatomical and histopathological features,^45^ and more accurately mimics the tumor’s ability to grow and metastasize.^46^ Orthotopic transplantation requires high technical manipulation in a specific anatomical location. Furthermore, in later stages, it may be necessary to monitor the tumor growth and specific location with non-invasive imaging techniques such as computed tomography or ultrasound.^47^^,^^48^ Thus, it may be technically challenging.

Compared with orthotopic transplantation, heterotopic transplantation is relatively simple.^22^ Tumor growth is commonly confined to the tumor implantation site, which is conducive to more accurate monitoring of tumor size and location, but it cannot simulate the real tumor microenvironment and tumor metastatic process.^44^ Currently, subcutaneous transplantation is the most used approach.^49^ However, studies have shown that non-subcutaneous transplantation provides better angiogenic capacity.^45^ Therefore, determining the optimal implantation site for various tumor types is essential for subsequent studies in the PDX model.

Meanwhile, in establishing and maintaining the PDX model, fresh tissue samples are usually preferred for transplantation, better than overnight stored or cryopreserved tumor fragments.^50^ Simultaneously, to maximize the preservation of tumor viability, the time gap between sample acquisition and implantation should be shortened as soon as possible, and fresh tumor samples should be kept in a cryogenic environment after isolation.^26^^,^^51^

However, due to cost and time limitations, frozen graft specimens are often used, especially during subsequent passaging.^51^^,^^52^ These tumor samples are generally stored in liquid nitrogen using specific cryopreservation agents.^40^ Alkema et al^53^ and Ivanics et al^54^ tested the effect of different tumor preservation protocols on cryopreservation and resuscitation activity of PDX tumors and showed that samples based on fetal calf serum/dimethyl sulfoxide^53^ and the use of specialized cryoprotectants^54^ improved post-resuscitation transplantation success rates.

## Implanted tumor type and malignancy

When modeling PDX, tumor types and their malignancy are important factors influencing the success rate. Tumors with high malignancy, metastasis, and invasiveness have a higher success rate.^55^ Also, Izumchenko et al implanted tumor samples from 1163 patients into immunodeficient mice, confirming significant differences in PDX implantation success rates among the different tumor types.^56^

Several studies have found that the success rate of PDX modeling is also correlated with patients' previous treatment history. For instance, Kuwata et al in constructing gastric cancer PDX models found that the implantation success rate of gastric cancer patients who received chemotherapy was higher than that of patients who did not receive chemotherapy.^57^ Meanwhile, Heo et al found that the success rate of epithelial ovarian cancer PDX models was negatively correlated with the overall survival rate of patients whose tumors were derived from epithelial ovarian cancer.^58^

**Figure 2:**
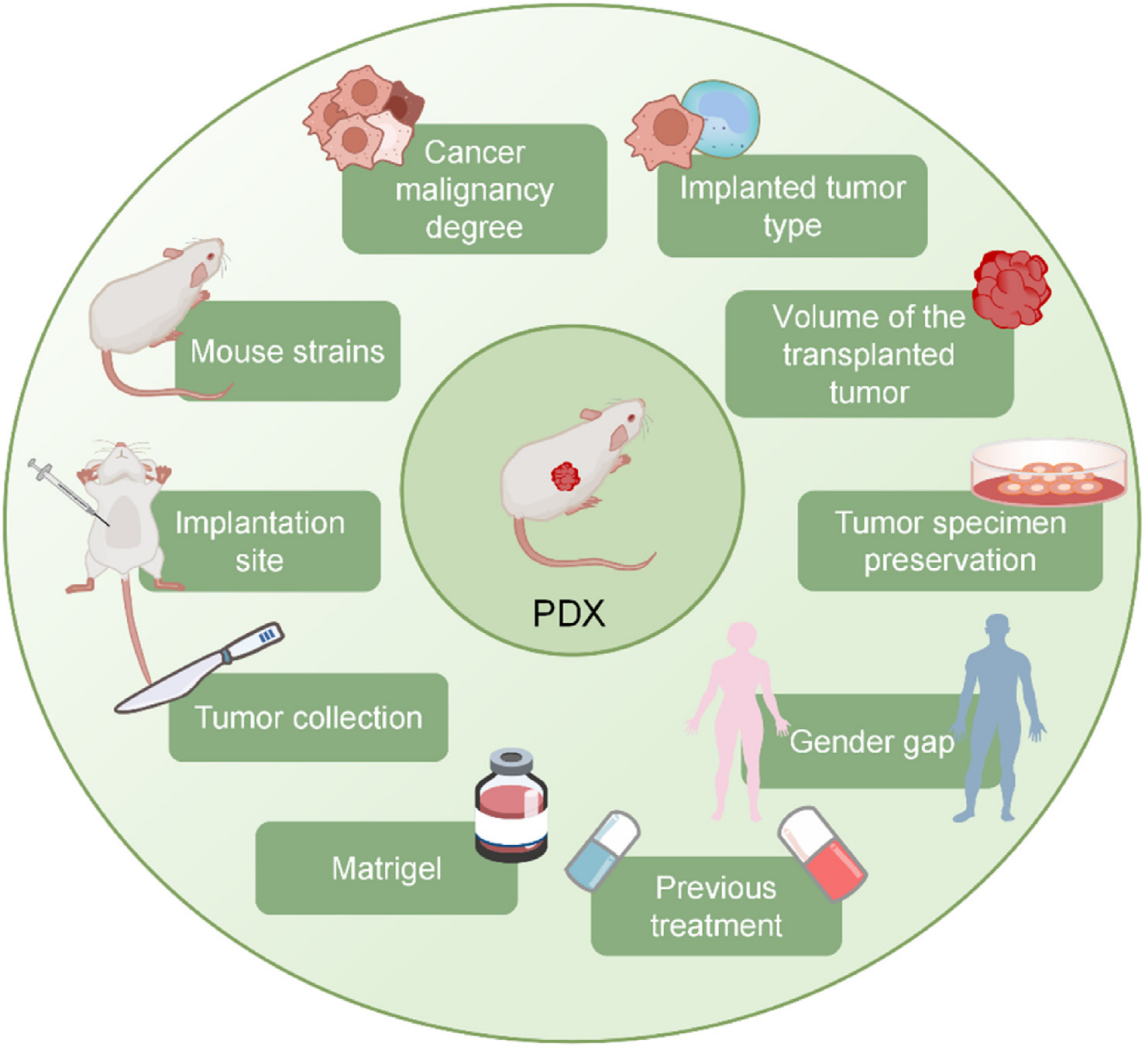
The distractors affecting the successful construction of PDX models.

In addition, studies have shown that for hormone-dependent tumors,^59^ such as most estrogen receptor-positive breast cancers and prostate cancers,^60^, ^61^, ^62^ by supplementing with relevant human hormones (*e.g.*, estradiol or testosterone), the success rate of PDX construction can be effectively improved.^24^ Besides, Liu et al found that PDX models of gastric cancer may be easier to establish in men than in women.^63^ This implies that among certain tumor types, gender differences may also affect PDX construction. Therefore, to maximize the value of PDX models, deeply understanding these influential factors is crucial for the efficient establishment of PDX models (Fig. 2).

## Advantages and limitations of PDX models

The xenograft models depending on their origin are categorized into cell line-derived xenograft models and PDX models.^64^ Cell line-derived xenograft models are constructed by transplanting *in vitro* cultured tumor cell lines into immunodeficient mice, which are currently one of the most commonly used preclinical models.^65^^,^^66^ However, *in vitro* cultured tumor cells are not fully characteristic of each cancer patient as they are generally derived from a specific tumor subpopulation.^40^^,^^67^ Additionally, during repeated passaging, tumor cell lines adapt to the *in vitro* culture environment, which will ultimately result in the original tumor genome expression and biomarker alterations,^47^^,^^68^ with poor predictive value for clinical drug development.^25^^,^^69^

However, the PDX model faithfully encapsulates the gene profile expression of transplanted tumors, preserving more than 80% genome of the original tumor tissue.^69^^,^^70^ It was reported that the PDX model was able to maintain genomic stability with high fidelity in the first 10 passages to ensure adequate experimental cycles.^40^^,^^71^ Simultaneously, PDX models preserve high-fidelity histological characteristics of transplanted tumors.^72^ Braekeveldt et al found that the neuroblastoma PDX model exhibited genomic, transcriptional, and phenotypic stability for more than two years through serial passaging and comprehensive molecular analyses.^73^ One study observed that in glioblastoma multiforme PDX models, mRNA expression levels of specific genes, such as cell-cycle modules, showed a stable transcriptional signature.^74^ Similarly, another study demonstrated that xenografts reflect specific markers of tumor histological subtypes at the mRNA level, despite the reduction of extracellular matrix.^75^ Moreover, although there was a 17% difference in miRNA expression between the patient tumors and the PDX model, it did not change significantly with prolonged PDX passaging.^76^ This indicates that the PDX model is more appropriate for the accurate identification of tumor-specific biomarkers for diagnostic, prognostic, and therapeutic targeting.

Furthermore, PDX models have shown consistency in simulating clinical drug responses.^26^ Similarly, several large-scale PDX clinical studies on drug response and resistance mechanisms have affirmed the reproducibility and translational clinical utility of PDX.^41^^,^^77^^,^^78^ Such evidence clearly indicates that PDX is a trustworthy preclinical model.

While PDX models are increasingly valued in cancer research, they indeed have limitations that should be clearly understood to achieve their optimal application. Firstly, the success rate of tumor engraftment is currently variable. For instance, tumors with poor patient prognosis often exhibit higher engraftment and metastatic capabilities.^79^ However, tumors of high malignancy and low differentiation may become more unstable after implantation.^72^^,^^76^ Secondly, developing a PDX model for preclinical use is time-consuming, while patient survival times are limited, and the development of models with low engraftment rates can further delay treatment plans and increase costs in clinic practices.^26^

Despite the application of advanced imaging technologies for tumor monitoring and visualization in PDX models, such as diffusion-weighted magnetic resonance imaging, dynamic contrast-enhanced magnetic resonance imaging, micro-computed tomography, and positron emission tomography tracer imaging,^52^^,^^80^, ^81^, ^82^ the implementation of these techniques in PDX models with maximal efficacy remains challenging. While PDX models preserve the histological structure of the original patient tumors, murine stromal components replace human stromal cells during passaging.^16^ These changes in the stromal components significantly affect tumor development by altering signaling pathways and gene expression profiles.^83^^,^^84^

Therefore, while the heterogeneity of the original tumor was shown to be generalized by PDX models, alterations in the tumor microenvironment result in the generation of different selective pressures to influence tumors' clonal evolution, rendering PDX models not entirely accurate to present the heterogeneity of patient tumors.^85^^,^^86^ Moreover, Sprouffske et al assessed the genetic heterogeneity in two triple-negative breast cancer PDXs,^85^ and whole-exome sequencing results suggested that mouse stroma could be a confounding factor for assessing tumor heterogeneity, allowing genetic heterogeneity to be exaggerated in the assessment.^30^^,^^85^ A study by Blomme et al in the colorectal cancer liver metastasis PDX model found that despite the early replacement of human stroma by mouse stroma, the PDX model remained somewhat stable at the metabolic level, implying that human cancer cells “educate” murine stromal cells to express a human-like phenotype during PDX development.^87^

Cross-contamination of cell lines and mouse viral infections are pervasive issues in cancer research, with new studies highlighting phenomena such as human cancer cell-to-murine oncogenic cell transformation and normal cell-to-cancer cell transformation in PDX models.^15^ Also, PDXs have detected xenotropic murine leukemia viruses^88^ and murine endogenous retroviruses.^89^^,^^90^ Once activated, these viruses can induce spontaneous tumor formation in mice (especially in the F1 generation^24^^,^^91^). Such uncontrollable factors introduce a multitude of uncertainties in various studies.

Admittedly, while the PDX model has demonstrated strong potential and reliability in preclinical studies, its limitations have somewhat constrained its broad application in precision medicine and personalized therapy. Therefore, to ensure the accuracy of personalized therapy, researchers are constantly searching for new methods and technologies to improve the PDX model, such as by continuously optimizing the construction process and evaluation methods of the PDX model, conducting detailed biological validation and comparison of clinical data, constructing diversified animal models (including the PDX mouse model), and actively integrating new technologies and methods to combine with the PDX model, *etc.*, which will more effectively guide the development of personalized treatment strategies, thereby improving the predictive ability and clinical application value of the PDX model.

## Innovation of PDX models

**Figure 3:**
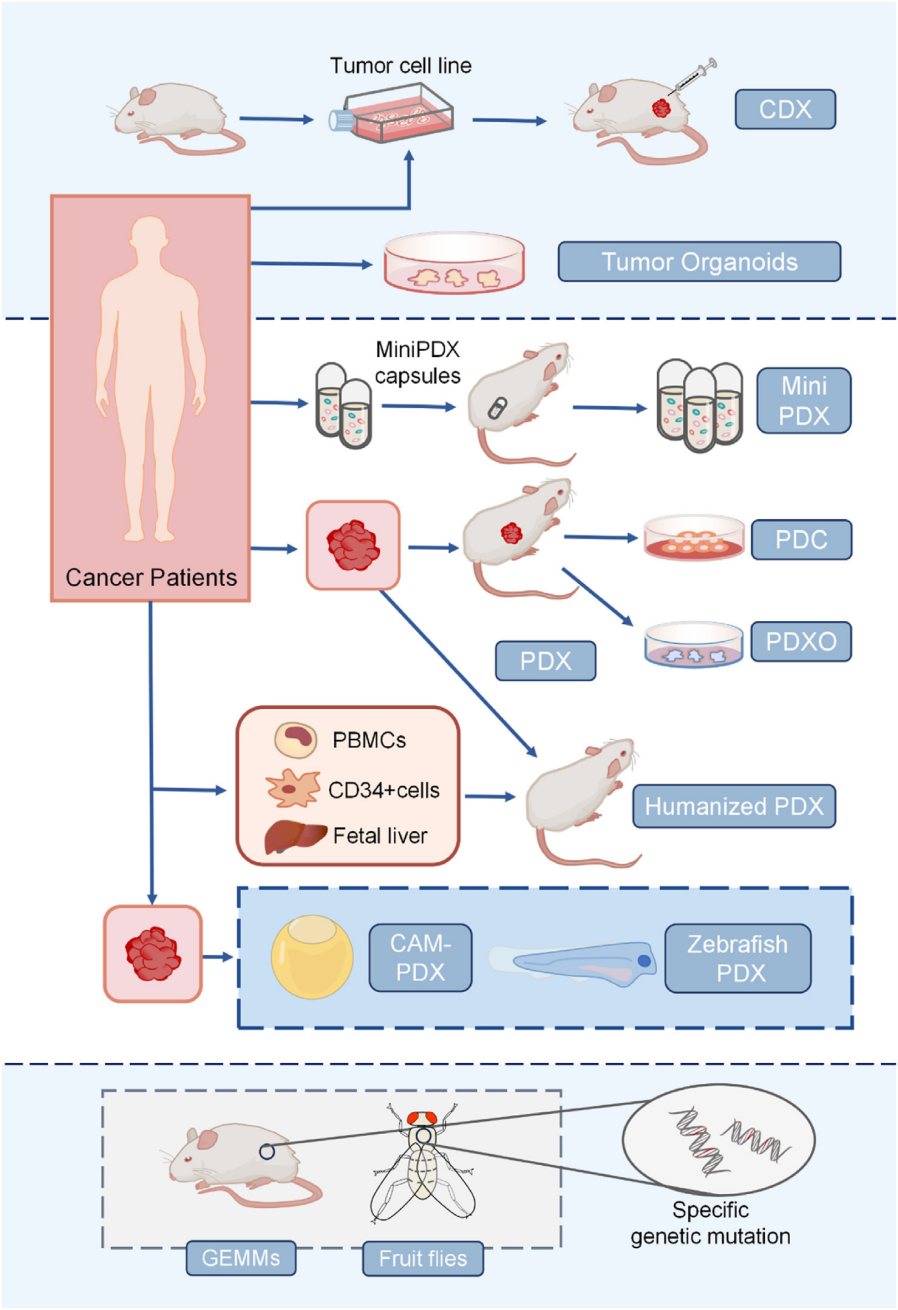
Schematic representation of different preclinical models. PDO, patient-derived organoids; Mini PDX, mini patient-derived xenograft; GEMM, genetically engineered mouse model; PDC, patient-derived cell cultures; CAM, chicken egg chorioallantoic membrane.

Recently, platforms like Xenograft Visualization & Analysis (Xeva) have emerged, offering researchers powerful tools for storing, accessing, visualizing, and analyzing complex pharmacogenomic data during *in vivo* drug screening.^68^ Additionally, combining high-throughput omics technologies (whole-genome sequencing, whole-exome sequencing, RNA sequencing, single-cell RNA sequencing), and bioinformatics methods, have effectively addressed the challenges faced by PDX models.^92^ To ensure the quality and reproducibility of PDX models, Meehan et al introduced the Minimum Information Standard for PDX Models (PDX-MI), defining the minimum information required for PDX models, including clinical details of patient tumors, mouse strains, and implantation methods.^93^ Although the advantages of PDX models in cancer research are becoming clear, as cancer studies advance, more appropriate preclinical models are increasingly important to develop (Fig. 3). They minimize the possibility of clinical trial failures while providing more effective personalized treatment options for cancer patients.

## Patient-derived xenograft cells

Compared with PDX models, PDX cells are less costly and easier to manipulate, suitable for high-throughput drug screening and *in vitro* gene regulation.^94^^,^^95^ Several studies have shown that PDX cells have a higher success rate than primary tissue-cultured cell line models, for one reason that human fibroblasts in PDX tissues are easily replaced by mouse fibroblasts,^12^^,^^94^ which are more sensitive to mechanical and enzymatic removal, with less time to eliminate.^12^^,^^96^ Concurrently, compared with traditional cell line models, PDX cells are better able to preserve the histology, molecular characteristics, and degree of tumor heterogeneity from the original tumor.^96^^,^^97^ However, cholangiocarcinoma research has found that PDX cell models may experience the loss of the Y chromosome during *in vitro* cultivation.^12^^,^^98^ This phenomenon alerted researchers to the lack of accuracy in maintaining gene expression in PDX cells. Nevertheless, PDX cells certainly provide a cellular resource closer to the patient’s original tumor traits for cancer research.^98^

## MiniPDX models

The advent of the MiniPDX has mitigated some limitations of PDX models and organoid models, such as the long modeling time and low success rate of PDX, and organoids' inability to fully assess drug responses.^99^ MiniPDXs are created by preparing a single-cell suspension from fresh tumor samples obtained from patients and encapsulating it in OncoVee capsules,^99^ which are then subcutaneously implanted into immunodeficient mice.^6^ This hollow fiber culture system enables rapid assessment of drug sensitivity within approximately seven days.^3^^,^^100^^,^^101^ Zhang et al showed that the MiniPDX model’s drug sensitivity test results exhibit up to 92% concordance with PDX models.^102^ Although MiniPDX still has limitations, such as not fully replicating the complex tumor microenvironment, its short testing cycle, low cost, and high concordance of drug response assessment with clinical treatment outcomes,^102^ making it a highly effective alternative to the PDX model, promising to accelerate the drug discovery process and optimize treatment strategies.

## Tumor organoids

Patient-derived organoids are miniature three-dimensional culture models constructed from patient tumor tissue or circulating tumor cells.^103^ The landmark significance of tumor-like organ models, which can be preserved, revived, and continuously passaged with self-renewal and self-propagation capabilities. They can rapidly summarize and maintain the original tumor’s genotypic features, histological phenotype, and heterogeneity,^103^ and allow gene-level editing and modifications,^104^ offering substantial opportunities for developing large biobanks.^105^^,^^106^

PDX-derived organoids are an innovative model. Xu et al successfully created a large repository of over 550 PDX-derived organoids, confirming their consistency with parent PDX in terms of drug response, molecular biological features, and genomic expression.^107^ The PDX-derived organoid model has been widely used for drug efficacy evaluation, targeted therapy, and the development of biomarkers.^108^ PDX-derived organoid models have also been employed to study tumor types that are relatively scarce in preclinical models, such as salivary gland cancer.^109^

While organoids show great application potential in cancer research, they still present several challenges including the absence of a vascular system in organoids, dependence on culture media for nutrients, size restriction, the lack of stromal cells (making it difficult to accurately replicate the tumor microenvironment), uncontrollable reproducibility and success rates, and high costs.^103^ In response to these challenges, researchers are striving to develop various innovative methods that integrate organoid models with other cutting-edge technologies, such as organ-on-chips, three-dimensional bioprinting, acoustic droplet printing, and CRISPR-Cas9 technology.^103^^,^^110^^,^^111^ As well, as an *in vitro* culture model, organoids are not fully representative of the actual tumor growth.^99^

## Humanized mice

Since immunodeficient mice are usually selected as hosts for PDX model construction, they lack an intact immune system and have limitations in immunological studies. Therefore, researchers developed the humanized mouse model with a human immune system.^112^ It originated in the late 1980s, and the humanized mouse model has been widely used in tumor research and become a powerful tool for immunotherapy research.^40^ Humanized mouse models can be categorized into three main types, human peripheral blood mononuclear cell mouse model,^109^ human hematopoietic stem cell mouse model,^110^ and bone marrow-liver-thymus mouse model.^113^

Humanized mice are created by transplanting human immune cells into mice. Subsequent transgenic humanization replaces mouse genes with corresponding human genes for construction purposes.^114^ Humanized PDX models built on the foundations of established humanized mice, then transplanting patient-derived tumor tissues into humanized mice.^115^ These models have become valuable tools for analyzing tumor-immune system interactions and evaluating immunotherapies, such as PD-1 (programmed death-1) targeted tumor immunotherapy and drug testing.^115^

While PDX models can undergo multiple generations, hematopoietic systems cannot and require frequent invasive sampling to establish individualized humanized PDX mouse models.^113^ Though these humanized models offer new avenues for researching cancer treatment strategies, there are inconsistencies in immune system reconstitution and other technical challenges, such as uncontrollable factors like the source and lifespan of patient hematopoietic cells.^24^^,^^116^ To enhance the standardization of humanized mouse models, researchers have proposed Minimal Information for Standardization of Humanized Mice (MISHUM) to ensure more systematic and reproducible humanization processes.^65^

## Genetically engineered mouse models

With the development of genetic engineering technology, genetically engineered mouse models have been introduced. These models involve the modification of mouse genes to construct tumor models, primarily using tissue-specific promoters to control the oncogenes expression or tissue-specific recombinases to promote tumor suppressor gene loss.^66^^,^^117^ The advantage of genetically engineered mouse models is that they allow for tumor growth in a mature immunological microenvironment in mice, complete with a full immune system and tumor environment.^66^^,^^118^

Genetically engineered mouse models can effectively mimic the entire carcinogenesis process from precancerous conditions to tumor development, accelerating cancer research from a genetic perspective. The Cre-loxP system is widely used to construct conditional genetic modification mouse models,^47^ but traditional Cre-loxP systems are not fully capable of replicating the sequential accumulation of mutations that occur during tumor formation.^117^ Consequently, Schönhuber et al have developed an inducible dual recombinase system that combines Flippase-FRT and Cre-loxP recombination techniques.^119^ CRISPR/Cas9 technology has become the preferred system for modeling genetically engineered mouse models with the ability to selectively delete specific gene profiles.^120^

Nevertheless, genetically engineered mouse models have limitations in mimicking human cancer genesis mechanisms. One key issue is that human tumors typically originate from the gradual accumulation of mutations in a small subset of cells, whereas genetically engineered mouse models influence changes in gene profiles in all cells.^66^^,^^121^ Tumor growth in genetically engineered mouse models has a longer latency period, due to incomplete consistency in the exogenous rate of mutations, which results in asynchronous tumorigenesis in different mice, implying a higher time cost and economic investment.^99^^,^^122^

Mouse models are the most used preclinical models in cancer research, however, due to their limitations, it is necessary for researchers to continuously develop more preclinical animal models to further advance the development of oncology research.

## Zebrafish PDX

In 2005, Lee et al transplanted melanoma cells into blastula-stage embryos of zebrafish,^123^ marking the initial confirmation of this model in cancer research. Subsequently, many researchers have chosen zebrafish as a new host for PDX models.^124^^,^^125^ Zebrafish share 70% genetic homology with humans, and at least one zebrafish orthologue exists for 82% of human disease-causing genes.^124^^,^^126^ The zebrafish model, based on the age at implantation, can be categorized into embryonic zebrafish, larval zebrafish (most used), and adult immunodeficient zebrafish.^18^

Compared with mouse PDX models, zebrafish PDX models enable complete systemic imaging on the whole animal and visualize drug responses and metastasis at the single-cell level.^127^ Zebrafish PDX models require smaller sample sizes and are cheaper and faster to construct, primarily due to their external fertilization and rapid reproduction and development.^122^

Despite these advantages, zebrafish PDX models have some drawbacks, including instability in implantation efficiency and high mortality rates.^128^ Additionally, differences in pharmacodynamic responses and drug metabolism exist between zebrafish and humans due to the zebrafish not being mammals, making it difficult to achieve drug conversion.^128^^,^^129^ Also, the typical rearing temperature for zebrafish is 28 °C–29 °C, but a compromise temperature of 34 °C has been identified by researchers.^130^

## Drosophila models

Apart from zebrafish PDX models, Bangi et al developed a genetically engineered drosophila model to screen for potential colorectal cancer treatment strategies.^131^ About 75% of human disease genes have homologues in drosophila.^132^ The advantages of the drosophila model include easier handling and higher efficiency compared with mouse models, allowing for the alteration of many genes within a single tissue and the high-throughput screening of promising anti-cancer drugs in less time,^133^ with a straightforward readout of efficacy and drug toxicity.^131^ Additionally, current researchers have developed humanized drosophila models,^134^ and future studies are expected to increasingly utilize drosophila models, despite their complexity which currently results in variable success rates.

Moreover, the chicken egg chorioallantoic membrane is an alternative model to mouse PDX, used to study tumor angiogenesis and as a model for screening anti-angiogenic drugs.^135^ Due to the chicken embryo’s lacking the functional immune system before 18 days, chorioallantoic membrane models are relatively easier to establish, but have shorter observation periods and are not well suited for studying tumor metastasis.^135^^,^^136^

As cancer research advances, some large animal cancer models have provided new platforms for researchers, addressing some of the limitations in small animal models, such as difficulty in performing complex surgical procedures.^101^ For instance, dog and pig animal models are more similar to human genomic profiles, anatomical structures, and physiological functions.^134^^,^^137^ Ressel and colleagues found a loss of PTEN protein expression in both canine mammary cancer and human breast cancer.^138^ Researchers have developed a Cre-inducible Cas9 expression gene-edited pig,^139^ and thereafter, Jin et al constructed a doxycycline-induced SpCas9 expression pig model,^140^ providing powerful tools for *in vivo* or *ex vivo* genome and epigenome editing in cancer research. Furthermore, Tu et al established a new model for pancreatic ductal adenocarcinoma in tree shrews.^141^

The development of new preclinical experimental platforms has undoubtedly provided a new direction for cancer research. It is evident that although each preclinical model has its advantages, it inevitably possesses certain limitations as well.

## Applications of PDX models in various types of cancer

**Figure 4:**
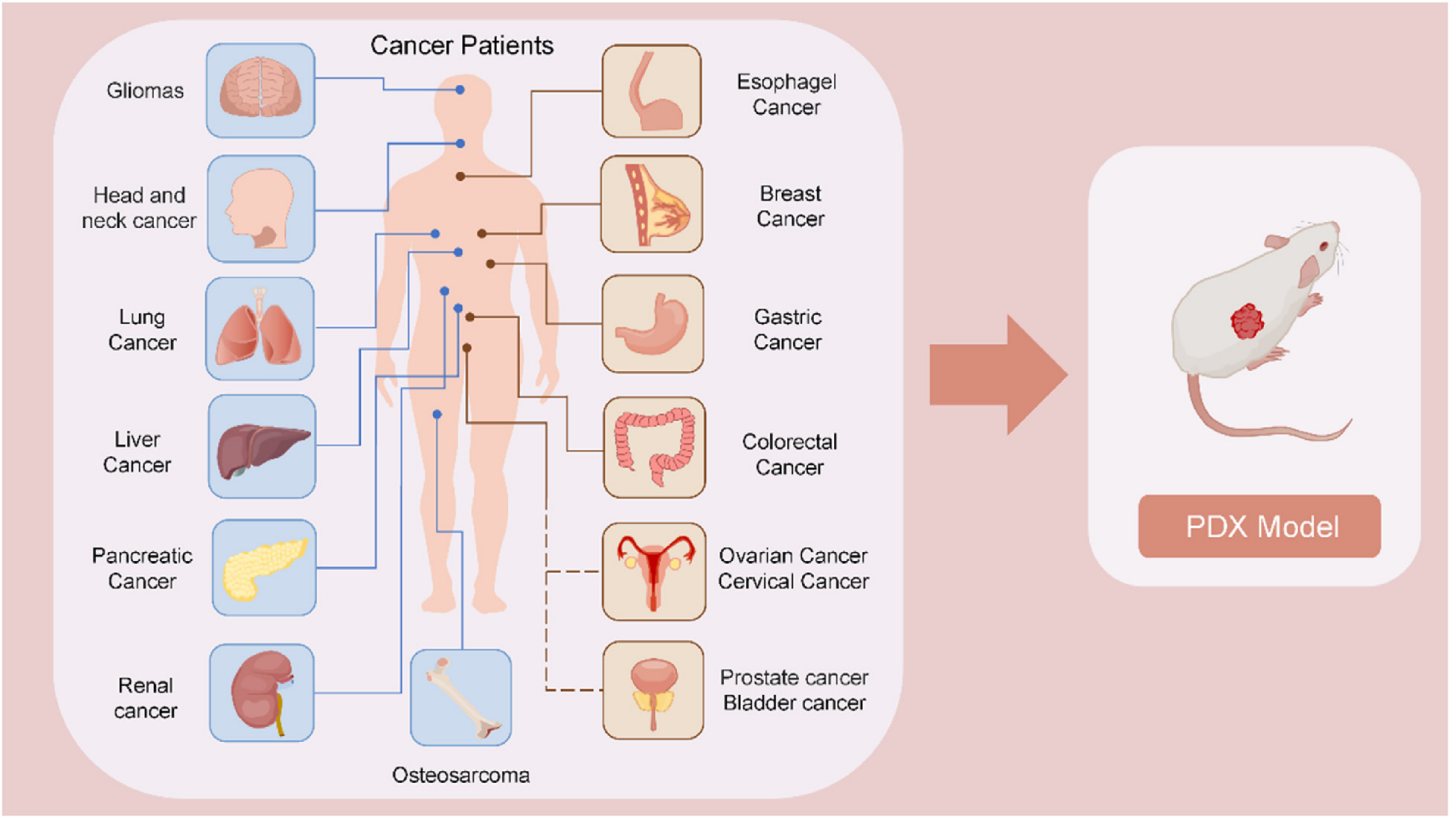
PDX models have been applied to multiple human cancer types.

The National Cancer Institute (NCI) has recommended PDX models to replace NCI-60 (Human Tumor Cell Lines) as a better model for drug screening.^142^ PDX model has been increasingly recognized as a more reliable preclinical platform and has been used in multiple research areas for a variety of tumor types. In the following, we review the diverse applications of PDX models as preclinical models in various types of cancer (Fig. 4).

## Lung cancer

Lung cancer originates from the bronchial mucosa or lung parenchyma, is a common, highly heterogeneous, and high-mortality type of malignant tumor, and remains the leading cause of cancer death globally.^1^^,^^143^ PDX models have been widely used for more accurate assessment of drug efficacy and drug screening, providing more personalized treatment options for lung cancer patients. Additionally, researchers have coupled PDX with CRISPR-Cas9 in the field of molecular targeted cancer therapy, demonstrating that pharmacological inhibition of the deubiquitinase USP7 can re-sensitize to chemotherapy in small cell lung cancer.^144^ There have been new constructions for lung cancer PDX, for example, Wang et al established a genetically edited lung cancer pig model,^139^ and Ali et al developed a combined mouse-zebrafish PDX platform for non-small cell lung cancer.^145^

## Gastrointestinal cancers

Gastrointestinal cancers are the most common type of cancer in the world and the disease with the highest morbidity and mortality rates.^146^ As more new technologies are being developed, the treatment of gastrointestinal tumors has significantly progressed, yet the need for precise treatment of most cancer patients has not been met, mainly due to insufficient and misleading preclinical data.^147^^,^^148^ Therefore, the PDX model has become a more reliable preclinical platform for gastrointestinal tumors due to its unique advantages.

Primary liver cancer is one of the sixth most common cancers in the world and can be histologically divided into hepatocellular carcinoma and intrahepatic cholangiocarcinoma.^3^^,^^6^ The hepatocellular carcinoma PDX model first came into the public in 1996.^149^ Afterward, PDX modeling has been widely used in liver cancer research, such as mechanism study and target prediction. A recent study that combined several preclinical models for hepatocellular carcinoma (tumor cell lines, cell line-derived xenograft, PDX, and patient-derived organoid) confirmed that diclofenac exerted anti-hepatocellular carcinoma effects by inhibiting NMT1 (N-myristoyl transferase 1)-mediated myristoylation of the VILIP3 protein.^150^ This finding suggests diclofenac as a potential novel anti-cancer drug and NMT1 as a possible therapeutic target for hepatocellular carcinoma.^150^ Intrahepatic cholangiocarcinoma research also benefits from PDX models, for instance, Huang et al used an intrahepatic cholangiocarcinoma PDX model to confirm the oncogenic role of YTHDF2 (YTH domain-containing family protein 2) and its contribution to cisplatin treatment desensitization.^151^ Furthermore, pediatric hepatocellular carcinoma is a rare but extremely low-survival tumor type in young children with some degree of differentiation from adult cancers, and the majority of pediatric liver cancer research has relied on single cancer cell lines, lacking preclinical animal models capable of precisely capturing their heterogeneity and metastatic potential. To address this gap, Bissig-Choisat et al established PDX models for pediatric liver cancer (including hepatoblastoma and hepatocellular carcinoma).^152^

Gastric cancer has a poor prognosis, ranking fifth globally in incidence and fourth in mortality with most gastric cancers diagnosed late.^149^ Ma et al developed two high-affinity and high-specificity human CDH17 nanobodies (A1 and E8), which showed significant anti-tumor effects in gastric cancer PDX (including mouse PDX and zebrafish PDX) when fused with imaging probes and toxins PE38,^153^ offering a promising new imaging modality and clinical translational therapy for gastric cancer treatment. Moreover, the construction of gastric cancer metastasis models has been a challenge in gastric cancer research, for which, some researchers have developed various gastric cancer metastasis models and new techniques and methods.^154^^,^^155^

Colorectal cancer is the third most common malignancy worldwide, affecting both men and women equally. The PDX implantation success rate for colorectal cancer is high.^56^ PDX models have shown significant value in identifying therapeutic targets for colorectal cancer treatment. Zheng et al illustrated the mechanisms of the PTEN-generated circRNA circPTEN1 in colorectal cancer metastasis,^156^ and Zeng et al revealed the regulatory role of circ-YAP-encoded oncoprotein YAP-220aa in patients with colorectal cancer with liver metastasis.^157^ Colorectal cancer PDXs have also been applied in various fields such as metabolic reprogramming epigenetics,^158^ drug screening,^159^ assessing drug resistance,^160^ and microbe-host relationships.^161^ These findings offer new perspectives for colorectal cancer treatment.

Esophageal cancer is classified into esophageal squamous cell carcinoma and esophageal adenocarcinoma based on their different cellular origins.^27^ Since early diagnosis of esophageal cancer poses a consistent challenge in clinical settings, screening for predictive biomarkers in esophageal cancer is essential for identifying early-stage esophageal cancer patients and implementing effective treatment strategies. Chu et al identified that TIGAR (TP53-induced glycolysis and apoptosis regulator) may be a predictive biomarker to guide esophageal squamous cell carcinoma treatment strategies in esophageal squamous cell carcinoma PDX.^162^ Another study highlighted the potential of NQO1 (NAD(P)H quinone dehydrogenase 1) as a biomarker for esophageal squamous cell carcinoma.^163^

According to global cancer statistics, the number of deaths from pancreatic cancer almost equals the number of cases.^164^ Pancreatic ductal adenocarcinoma, the most common type of pancreatic cancer, has an extremely poor prognosis and frequently metastasizes to the liver, peritoneum, and lungs.^150^ Currently, PDXs are extensively used in pre-clinical research for pancreatic ductal adenocarcinoma. Moreover, Stossel et al investigated the novel humanized germline BRCA-associated pancreatic ductal adenocarcinoma PDX in predicting therapeutic responses.^165^

## Breast cancer

In 1903, researchers first successfully established a transplant model for breast cancer cell lines. A century later, in 2003, the effective modeling of human tumor tissue was realized by the implantation of immunodeficient mice.^166^ Currently, PDX models are applied in diverse breast cancer research efforts. Additionally, researchers are combining breast cancer PDX models with PDX-derived organoids to assess drug efficacy, resistance, and combined clinical treatments.^167^ These include evaluating the therapeutic effects of CDK4/6 (cyclin-dependent kinase 4/6) inhibitors combined with endocrine therapy^168^ and testing the regulatory and anti-tumor effects of DOT1L (disruptor of telomeric silencing-1-like) inhibitors on triple-negative breast cancer stem cells in triple-negative breast cancer PDX and PDX-derived organoid.^169^

## Gynecologic tumors

Gynecologic tumors are complex in type, and although the available treatment strategies continue to improve (including aggressive surgical treatments and platinum-based chemotherapy),^170^ patient survival remains low due to the presence of chemotherapy resistance and risks associated with tumor recurrence during the treatment process.

Ovarian cancer is considered the deadliest among gynecological cancers. Despite initial responsiveness to platinum-based therapies, many patients are at risk for recurrence or development of resistance to treatment, especially in cases of high-grade serous ovarian cancer.^52^^,^^171^ Whether as a monotherapy or in combination with chemotherapy, PARP (poly ADP-ribose polymerase) inhibitors have shown great therapeutic potential in the treatment of ovarian cancer.^172^ However, PARP inhibitors and platinum resistance are also major clinical challenges in ovarian cancer.^173^ Zhou et al used the PDX model and discovered that the antibiotic novobiocin could be used alone or in combination with PARP inhibitor for homologous recombination-deficient breast or ovarian cancer.^174^ Subsequent new studies have confirmed that mifepristone enhanced ovarian cancer treatment response and overcame (PARP inhibitor) olaparib resistance by targeting polyploid giant cancer cells.^175^ Other studies have identified new therapeutic targets for ovarian cancer that may address resistance to platinum agents or PARP inhibitors, such as phosphoglycerate dehydrogenase (PHGDH)^173^ and master regulators of mitochondria (PGC1α/β).^176^

Cervical cancer is a common malignancy among women, despite various preventive measures available, such as the human papillomavirus vaccine, early screening, and treatment options, the outcomes for metastatic or recurrent cervical cancer remain suboptimal.^52^^,^^164^ Furthermore, some preventive measures have not been equitably implemented worldwide. Currently, studies have been conducted to generalize the available cervical cancer PDX models to identify the most appropriate methods for constructing cervical cancer PDX and to better use them for subsequent preclinical studies.^177^ For example, Liu et al constructed the largest cervical cancer PDX biobank to date and assessed the combined effect of neratinib and adoptive cell therapy on patients with HER2 (human epidermal growth factor receptor 2)-mutated cervical cancer.^178^

Further, there are rarer types of female cancers such as vulvar and vaginal cancers,^170^ which often exhibit unique molecular features and require further development of new effective targets.

## Pediatric solid tumors

Research into pediatric malignancies has revealed the heterogeneity and molecular changes that set children’s solid tumors apart, which account for 60% of all pediatric cancers. Unlike adult cancers, children’s cancers necessitate the development of specialized diagnostic and treatment approaches.^179^

Neuroblastoma, originating in the developing peripheral sympathetic nervous system, is a common solid tumor in infants and early childhood.^180^ An early attempt at neuroblastoma xenograft modeling was conducted by Tsuchida et al in 1984, who transplanted human neuroblastoma into nude mice to assess the effects of chemotherapy drugs and surgical treatment on tumor growth and viability.^181^ High-risk neuroblastoma accounts for about 15% of all pediatric cancer deaths,^182^ and MYCN oncogene amplification is present in over 30% of high-risk neuroblastomas.^183^ Since neuroblastomas are often associated with genomic mutations, the genetically engineered mouse model shows great potential in neuroblastomas, whereas the PDX model incorporates more complexities and is better suited for personalized treatment.^184^ Therefore, a study has used *in vitro* (tumor cell lines, patient-derived cell cultures) and *in vivo* (genetically engineered mouse models and PDXs) neuroblastoma models to assess the treatment efficacy of combining the ALK (anaplastic lymphoma kinase) inhibitor lorlatinib with the MDM2 (murine double minute clone 2) inhibitor (idasanutlin) for ALK-aberrant neuroblastoma.^185^

Osteosarcoma is a rare and highly aggressive mesenchymal malignant primary bone tumor, accounting for less than 0.2% of all malignancies.^94^ Pascual-Pasto et al constructed PDX models of Ewing sarcoma, rhabdomyosarcoma, and osteosarcoma and found that pediatric solid tumors expressing SPARC (secreted protein acidic and cysteine-rich) accumulated albumin-bound paclitaxel over an extended period.^186^ Chen et al developed a nanoparticle, TGIC-CA (TC), and found that combining TC/miR-22 with volasertib synergistically inhibited the PI3K (phosphoinositide 3-kinase)/Akt (protein kinase B) signaling pathway for anti-osteosarcoma effects.^187^ These findings demonstrate the significant relevance and value of PDX models in pediatric solid tumor research, providing precision treatment guidance even in rare tumors such as osteosarcoma.^188^

## Prostate cancer

Prostate cancer is the second most common malignancy in men.^164^ Various preclinical prostate cancer cell line models have been developed to elucidate the complex therapeutic mechanisms in prostate cancer.^189^ However, nowadays, PDX models are increasingly used in prostate cancer research as an alternative to or in conjunction with two-dimensional cell culture models.^190^ To further optimize treatment strategies for prostate cancer research, researchers have also developed an MDA prostate cancer PDX model that effectively captures the clinical features of prostate cancer.^191^ Researchers have so far created numerous prostate cancer PDX models for practical prostate cancer research, for instance, predictive therapeutic targets,^192^ targeted therapy,^193^ and immunotherapy.^194^

## Bladder cancer

Bladder cancer is the tenth most common cancer worldwide, but the sixth most common cancer in men, as the incidence and mortality rates are four times higher in men than in women.^164^ Many researchers have established bladder cancer PDX models for tumor drug screening^195^ and the effects of drug combinations.^196^ Moreover, lymphatic metastasis is positively associated with poor prognosis in bladder cancer patients. An et al described the regulatory role of a novel intron-retaining circNCOR1 in bladder cancer lymph node metastasis,^197^ and another study confirmed the mechanism of action of HSF1 (heat shock factor 1) in bladder cancer lymphatic metastasis.^198^

As a key player in preclinical models, the PDX model provides an excellent platform for better understanding tumorigenesis, progression, and metastasis mechanisms, developing potential drug therapeutic targets, drug screening, designing personalized treatment regimens, and clinical combination therapy (Table 3). Besides the cancer types mentioned above, PDX models are also used for head and neck cancer,^199^ renal cell carcinoma,^200^ gliomas,^201^ *etc*.

**Table 3:** Potential therapeutic agents and their targets in different cancer types in PDX models.

Tumor type		Animal strain	Implantation site	Gene target	Drug	Application	Reference
Lung cancer	EGFR-mutant NSCLC	NSG mice	Subcutaneous, subrenal capsule	HER3	Osimertinib, HER3-DXd	Evaluation of combination therapy with osimertinib and HER3-DXd	Haikala et al^202^
	SCLC	NSG mice	Subcutaneous	UBA1	TAK-243	Evaluation of TAK-243 as mono- and combination therapy in SCLC	Majeed et al^203^
Gastric cancer		Balb/c-nu mice	Subcutaneous	JAK2, STAT3	CYT997	Confirmation that CYT997 may be a potential anti-tumor drug	Cao et al^204^
		NOD/SCID mice	Subcutaneous	CCAT5	si-CCAT5, oxaliplatin	Uncovering the mechanism of STAT3 signaling regulated by wnt signaling	Liu et al.^205^
Colorectal cancer		NOD/SCID mice	Subcutaneous	PIM1, FGFR1	HCI-48	Describing the anti-tumor effects of HCI-48 on the dual targeting of PIM1 and FGFR1	Yin et al^206^
		NOD/SCID mice	Subcutaneous	GART	Pemetrexed	Evidence for the function of GART and the role of the GART/RUVBL1/β-catenin signaling axis in promoting colorectal cancer stemness	Tang et al^207^
Esophageal carcinoma	ESCC	NOD/SCID mice	Subcutaneous	eEF2	Toosendanin	Revealed eEF2 as a potential therapeutic target for ESCC	Jia et al^208^
Liver cancer	Hepatoblastoma	Nude mice	Subcutaneous	ALCD	Olaparib	Explained the regulatory mechanism of ALCD in hepatoblastoma	Johnston et al^209^
	HCC	NOD/SCID mice	Liver	cDCBLD2, TOP2A	Sorafenib	Provides a potential strategy for targeting cDCBLD2 or TOP2A to overcome sorafenib resistance in patients with HCC	Ruan et al^210^
Pancreatic cancer	PDAC	Balb/c-nu mice	Orthotopic, intrasplenic	CD73	Diclofenac	Diclofenac may be an effective treatment for metastatic PDAC	Liu et al^211^
Breast cancer	Breast cancer	NSG mice	Mammarian fat pad	FGFRs	AZD4547, BLU9931	The potential of specific FGFRs as precision therapeutic targets was identified	Chew et al^212^
	ER-negative postmenopausal breast cancer	NSG mice	Mammarian fat pad	RANK	Denosumab	RANK protein expression is an independent biomarker of poor prognosis in patients with estrogen receptor-negative postmenopausal breast cancer	Ciscar et al^213^
Ovarian cancer	HGSOC	NOD/SCID mice	Subcutaneous	IGFBP2	Gold nanoparticles	Reported key signaling axes for gold nanoparticles' therapeutic role	Hossen et al^214^
	TP53 mutant ovarian cancer	Nude mice	Subcutaneous	IRE1α	AZD1775	Mechanism of UPR signaling network IRE1α in TP53 mutant ovarian cancer	Xiao et al^215^
Cervical cancer	Cervical cancer	NOD/SCID mice	Subcutaneous	ZNF275	Triciribine, cisplatin	ZNF275 is revealed to be a potential predictor of cervical cancer treatment	Ye et al^216^
Neuroblastoma	High-risk neuroblastoma	NMRI nude mice, NSG mice	Subcutaneous, adrenal glands	KSP	ARRY-520	KSP inhibition may be a promising treatment strategy for neuroblastoma	Hansson et al^217^
Osteosarcoma	Chemotherapy-resistant and metastatic osteosarcoma	NSG mice	Subcutaneous	β-catenin/ALDH1	Tegavivint	Evaluate tegavivint in chemotherapy-resistant and metastatic osteosarcoma in chemotherapy-resistant and metastatic osteosarcoma	Nomura et al^218^
Prostate cancer	NEPC	SCID mice	Subcutaneous	MYCN, CDK5, RB1, E2F1	Enzalutamide, olaparib, dinaciclib	Elucidating the mechanism of action for PARP inhibition in the treatment of NEPC	Liu et al^219^
Bladder cancer		NSG mice	Subcutaneous	ErbB3	Seribantumab	ErbB3 phosphorylation may be a potential therapeutic strategy for bladder cancer	Steele et al^220^
Renal cell carcinoma		NSG mice	Subcutaneous	ERK	Cabozantinib, sapanisertib	The potential of the combination therapeutic approach of cabozantinib and sapanisertib was emphasized	Wu et al^221^
HNC	HNSCC	Nude mice	Subcutaneous	RAC1, RAC3	EHOP-016, cetuximab	Uncovering biomarkers and mechanisms to overcome acquired cetuximab resistance	Yao et al^222^
Gliomas	Glioblastoma	Balb/c-nu mice	Mouse brain	PTRF	Temozolomide	Revealed PTRF as a biomarker for the prognosis of glioblastoma patients after temozolomide treatment	Yang et al^223^

Note: ESCC, esophageal squamous cell carcinoma; SCLC, small cell lung cancer; HCC, hepatocellular carcinoma; PDAC, pancreatic ductal adenocarcinoma; HGSOC, high-grade serous ovarian cancer; IGFBP2, insulin growth factor binding protein 2; IRE1α, inositol-required enzyme 1α; NEPC, neuroendocrine prostate cancer; HNC, head and neck cancer; HNSCC, head and neck squamous cell carcinomas; HER3, human epidermal growth factor receptor 3; UBA1, ubiquitin-like modifier activating enzyme 1; Jak2, Janus kinase 2; STAT3, signal transducer and activator of transcription 3; CCAT5, colon cancer-associated transcript 5; FGFR1, fibroblast growth factor receptor 1; PIM1, moloney-murine leukemia 1; GART, glycinamide ribonucleotide transformylase; RUVBL1, RuvB like AAA ATPase 1; eEF2, eukaryotic elongation factor-2; AICD, amyloid precursor protein intra-cellular domain; TOP2A, type IIA topoisomerase; DCBLD2, discoidin, CUB, and LCCL domain-containing protein 2; CD73, cluster of differentiation 73; RANK, receptor activator of nuclear factor-κB; ZNF275, zinc finger protein 275; KSP, kidney-specific cadherin; ALDH1, aldehyde dehydrogenase 1; CDK5, cyclin-dependent kinase 5; RB1, retinoblastoma protein 1; E2F1, E2F transcription factor 1; ERBB3, Erb-b2 receptor tyrosine kinase 3; ERK, extracellular signal-regulated kinase; RAC1/3, Ras-related C3 botulinum toxin substrate 1/3; PTRF, polymerase I and transcript release factor.

## Discussion

Construction and selection for preclinical models are crucial in cancer research, the most classical and widely used are tumor cell line models. However, single-cell line models have limitations in cell types and difficulty in generalizing the original tumor’s three-dimensional spatial organization. Under this background, researchers have developed xenografted *in vivo* animal models, namely cell line-derived xenograft and PDX models. The establishment of more complex *in vivo* animal models marks an advanced step in cancer research that overcomes the limitations of traditional two-dimensional culture. Especially the PDX model not only faithfully summarizes key features underlying the original tumors but also demonstrates immense potential in the development of anti-cancer drugs, clinical combination therapy, and designing personalized treatment strategies.

Mouse models, as the preferred animal model and research hotspot, have an unshakeable importance in cancer research history. To accommodate different research fields, scientists have developed various mouse strains. However, the construction of a PDX model is a complex process influenced by numerous factors, and slight variations at any step can significantly affect the eventual success of the model. Therefore, researchers must understand these variables deeply, draw continuously from existing experiences, renew technologies, and accumulate knowledge to optimize the model construction process and improve its success rate. While PDX models have been recognized as one of the most reliable preclinical models due to their excellent performance in the cancer field, it is also important to realize the limitations of the PDX model to achieve its optimal application and to develop a modeling platform more suitable for future cancer research.

The cultivation period for PDX models is relatively long and not applicable to medium–high throughput drug screening. To resolve these problems, scientists developed patient-derived organoid models, PDX-derived organoid models, and PDX cell models, which facilitate faster model construction and effectively recapitulate the molecular characteristics and drug responses of the original tumors. Furthermore, MiniPDX provides a faster way to achieve drug sensitivity assays. New animal models are emerging that offer more new possibilities and research platforms for cancer research. With the rapid development of genetic engineering technologies, genetically engineered mouse models and drosophila models are widely used in cancer research. Researchers developed a humanized PDX model based on humanized mice that could better simulate interactions between the tumor microenvironment and the immune system. Beyond mouse models, alternative models such as drosophila PDXs, zebrafish PDXs, chorioallantoic membrane PDXs, and large animal models including canines, pigs, and tree shrews, are becoming potential substitutes for mouse models.

Researchers are combining PDX models by actively utilizing new technologies such as TRANSACT (a computational framework that helps construct drug response predictors that robustly transfer from preclinical models to human tumors),^224^ DRAP (the first integrated toolbox for drug response analysis and visualization tailored for PDX platform),^225^ next-generation sequencing, fluorescence-activated cell sorting, genomics, and multi-omics,^40^ to optimize the construction of PDX models and better translate preclinical data for clinical use. Furthermore, new methods have been employed to standardize and evaluate the fidelity of different model types, such as PDX-MI, MISHUM, and CancerCellNet (CCN).^5^ Moreover, PDX models have demonstrated their value in research for various tumor types. Despite demonstrating significant potential in cancer research, PDX models have yet to achieve widespread breakthroughs in clinical practice. To facilitate the deeper development of PDX models, future research efforts should focus on several key aspects: optimizing the PDX modeling process, carefully selecting and validating animal models, developing biomarkers for predicting drug efficacy effectively, and exploring the combined application of new technological approaches, *etc*. These efforts aim to further improve the efficiency of preclinical data translation. Meanwhile, it is crucial to promote global data sharing and standardization of PDX models, which will greatly enhance the reliability and validity of its clinical application. Currently, PDX models are still under continuous refinement, but without a doubt, it offers a broad prospect as a highly promising preclinical experimental platform for the realization of precise and personalized cancer treatment.

## Funding

This work was supported by the 10.13039/501100001809National Natural Science Foundation of China (No. 82172653), the Intra Institutional Open Fund of School of Medicine, Hunan Normal University (No. KF2022001), the Key Project of Developmental Biology and Breeding from Hunan Province, China (No. 2022XKQ0205), and The Research Team for Reproduction Health and Translational Medicine of Hunan Normal University (No. 2023JC101).

## CRediT authorship contribution statement

**Min qi Liu:** Writing – original draft, Writing – review & editing. **Xiaoping Yang:** Conceptualization, Writing – review & editing.

## Conflict of interests

The authors declared no competing interests.

## References

[1] Siegel R.L., Miller K.D., Fuchs H.E., Jemal A. (2022). Cancer statistics, 2022. CA A Cancer J Clin.

[2] Sengupta R., Zaidi S.K. (2021). AACR cancer progress report 2021: discovery science driving clinical breakthroughs. Clin Cancer Res.

[3] Zhou Y., Xia J., Xu S. (2023). Experimental mouse models for translational human cancer research. Front Immunol.

[4] Luo J., Solimini N.L., Elledge S.J. (2009). Principles of cancer therapy: oncogene and non-oncogene addiction. Cell.

[5] Genta S., Coburn B., Cescon D.W., Spreafico A. (2022). Patient-derived cancer models: valuable platforms for anticancer drug testing. Front Oncol.

[6] Pan B., Wei X., Xu X. (2022). Patient-derived xenograft models in hepatopancreatobiliary cancer. Cancer Cell Int.

[7] Invrea F., Rovito R., Torchiaro E., Petti C., Isella C., Medico E. (2020). Patient-derived xenografts (PDXs) as model systems for human cancer. Curr Opin Biotechnol.

[8] Zanella E.R., Grassi E., Trusolino L. (2022). Towards precision oncology with patient-derived xenografts. Nat Rev Clin Oncol.

[9] Yoshida G.J. (2020). Applications of patient-derived tumor xenograft models and tumor organoids. J Hematol Oncol.

[10] Kirschbaum A., Geisse N.C., Judd S.T., Meyer L.M. (1950). Effect of certain folic acid antagonists on transplanted myeloid and lymphoid leukemias of the F strain of mice. Cancer Res.

[11] Toolan H.W. (1951). Successful subcutaneous growth and transplantation of human tumors in X-irradiated laboratory animals. Proc Soc Exp Biol Med.

[12] Okada S., Vaeteewoottacharn K., Kariya R. (2019). Application of highly immunocompromised mice for the establishment of patient-derived xenograft (PDX) models. Cells.

[13] Rygaard J., Povlsen C.O. (1969). Heterotransplantation of a human malignant tumour to “Nude” mice. Acta Pathol Microbiol Scand.

[14] Li X., Zhu D., Li N., Yang H., Zhao Z., Li M. (2017). Characterization of ascites-derived tumor cells from an endometrial cancer patient. Cancer Sci.

[15] Jin J., Yoshimura K., Sewastjanow-Silva M., Song S., Ajani J.A. (2023). Challenges and prospects of patient-derived xenografts for cancer research. Cancers.

[16] Roscilli G., De Vitis C., Ferrara F.F. (2016). Human lung adenocarcinoma cell cultures derived from malignant pleural effusions as model system to predict patients chemosensitivity. J Transl Med.

[17] Katsiampoura A., Raghav K., Jiang Z.Q. (2017). Modeling of patient-derived xenografts in colorectal cancer. Mol Cancer Therapeut.

[18] Wetterauer C., Vlajnic T., Schüler J. (2015). Early development of human lymphomas in a prostate cancer xenograft program using triple knock-out immunocompromised mice. Prostate.

[19] Hernandez M.C., Bergquist J.R., Leiting J.L. (2019). Patient-derived xenografts can be reliably generated from patient clinical biopsy specimens. J Gastrointest Surg.

[20] Oshi M., Okano M., Maiti A. (2020). Novel breast cancer brain metastasis patient-derived orthotopic xenograft model for preclinical studies. Cancers.

[21] Lai Y., Wei X., Lin S., Qin L., Cheng L., Li P. (2017). Current status and perspectives of patient-derived xenograft models in cancer research. J Hematol Oncol.

[22] Schuch L.F., Silveira F.M., Wagner V.P. (2020). Head and neck cancer patient-derived xenograft models - a systematic review. Crit Rev Oncol Hematol.

[23] Williams S.A., Anderson W.C., Santaguida M.T., Dylla S.J. (2013). Patient-derived xenografts, the cancer stem cell paradigm, and cancer pathobiology in the 21st century. Lab Invest.

[24] Zeng M., Ruan Z., Tang J. (2023). Generation, evolution, interfering factors, applications, and challenges of patient-derived xenograft models in immunodeficient mice. Cancer Cell Int.

[25] Chen K., Zhao H., Shi Y. (2019). Perioperative dynamic changes in circulating tumor DNA in patients with lung cancer (DYNAMIC). Clin Cancer Res.

[26] Jung J., Seol H.S., Chang S. (2018). The generation and application of patient-derived xenograft model for cancer research. Cancer Res Treat.

[27] Lan T., Xue X., Dunmall L.C., Miao J., Wang Y. (2021). Patient-derived xenograft: a developing tool for screening biomarkers and potential therapeutic targets for human esophageal cancers. Aging.

[28] Morton C.L., Houghton P.J. (2007). Establishment of human tumor xenografts in immunodeficient mice. Nat Protoc.

[29] Kurtz K.J., Conneely S.E., O’Keefe M., Wohlan K., Rau R.E. (2022). Murine models of acute myeloid leukemia. Front Oncol.

[30] Consortium M.G.S., Waterston R.H., Lindblad-Toh K. (2002). Initial sequencing and comparative analysis of the mouse genome. Nature.

[31] Zheng-Bradley X., Rung J., Parkinson H., Brazma A. (2010). Large scale comparison of global gene expression patterns in human and mouse. Genome Biol.

[32] Flanagan S.P. (1966). ‘Nude’, a new hairless gene with pleiotropic effects in the mouse. Genet Res.

[33] Makino S., Kunimoto K., Muraoka Y., Mizushima Y., Katagiri K., Tochino Y. (1980). Breeding of a non-obese, diabetic strain of mice. Jikken Dobutsu.

[34] Bosma G.C., Custer R.P., Bosma M.J. (1983). A severe combined immunodeficiency mutation in the mouse. Nature.

[35] Ogata-Aoki H., Higashi-Kuwata N., Hattori S.I. (2018). Raltegravir blocks the infectivity of red-fluorescent-protein (mCherry)-labeled HIV-1_JR-FL_ in the setting of post-exposure prophylaxis in NOD/SCID/Jak3^−/−^ mice transplanted with human PBMCs. Antivir Res.

[36] Ma X.L., Shen M.N., Hu B. (2019). CD73 promotes hepatocellular carcinoma progression and metastasis via activating PI3K/AKT signaling by inducing Rap1-mediated membrane localization of P110β and predicts poor prognosis. J Hematol Oncol.

[37] Piskounova E., Agathocleous M., Murphy M.M. (2015). Oxidative stress inhibits distant metastasis by human melanoma cells. Nature.

[38] Traggiai E., Chicha L., Mazzucchelli L. (2004). Development of a human adaptive immune system in cord blood cell-transplanted mice. Science.

[39] Ono A., Hattori S., Kariya R. (2011). Comparative study of human hematopoietic cell engraftment into BALB/c and C57BL/6 strain of rag-2/jak3 double-deficient mice. J Biomed Biotechnol.

[40] Liu Y., Wu W., Cai C., Zhang H., Shen H., Han Y. (2023). Patient-derived xenograft models in cancer therapy: technologies and applications. Signal Transduct Targeted Ther.

[41] Hidalgo M., Amant F., Biankin A.V. (2014). Patient-derived xenograft models: an emerging platform for translational cancer research. Cancer Discov.

[42] Taghian A., Budach W., Zietman A. (1993). Quantitative comparison between the transplantability of human and murine tumors into the subcutaneous tissue of NCr/Sed-nu/nu nude and severe combined immunodeficient mice. Cancer Res.

[43] Bondarenko G., Ugolkov A., Rohan S. (2015). Patient-derived tumor xenografts are susceptible to formation of human lymphocytic tumors. Neoplasia.

[44] Kang W., Maher L., Michaud M. (2021). Development of a novel orthotopic gastric cancer mouse model. Biol Proced Online.

[45] Braekeveldt N., Wigerup C., Gisselsson D. (2015). Neuroblastoma patient-derived orthotopic xenografts retain metastatic patterns and geno- and phenotypes of patient tumours. Int J Cancer.

[46] Hoffman R.M. (2015). Patient-derived orthotopic xenografts: better mimic of metastasis than subcutaneous xenografts. Nat Rev Cancer.

[47] Singhal S.S., Garg R., Mohanty A. (2023). Recent advancement in breast cancer research: insights from model organisms-mouse models to zebrafish. Cancers.

[48] Kim M.P., Evans D.B., Wang H., Abbruzzese J.L., Fleming J.B., Gallick G.E. (2009). Generation of orthotopic and heterotopic human pancreatic cancer xenografts in immunodeficient mice. Nat Protoc.

[49] Fu X., Guadagni F., Hoffman R.M. (1992). A metastatic nude-mouse model of human pancreatic cancer constructed orthotopically with histologically intact patient specimens. Proc Natl Acad Sci U S A.

[50] Tanaka T., Nishie R., Ueda S. (2022). Endometrial cancer patient-derived xenograft models: a systematic review. J Clin Med.

[51] Tracey A.T., Murray K.S., Coleman J.A., Kim K. (2020). Patient-derived xenograft models in urological malignancies: urothelial cell carcinoma and renal cell carcinoma. Cancers.

[52] Jiang W., Xie S., Liu Y., Zou S., Zhu X. (2020). The application of patient-derived xenograft models in gynecologic cancers. J Cancer.

[53] Alkema N.G., Tomar T., Duiker E.W. (2015). Biobanking of patient and patient-derived xenograft ovarian tumour tissue: efficient preservation with low and high fetal calf serum based methods. Sci Rep.

[54] Ivanics T., Bergquist J.R., Liu G. (2018). Patient-derived xenograft cryopreservation and reanimation outcomes are dependent on cryoprotectant type. Lab Invest.

[55] Weroha S.J., Becker M.A., Enderica-Gonzalez S. (2014). Tumorgrafts as *in vivo* surrogates for women with ovarian cancer. Clin Cancer Res.

[56] Izumchenko E., Paz K., Ciznadija D. (2017). Patient-derived xenografts effectively capture responses to oncology therapy in a heterogeneous cohort of patients with solid tumors. Ann Oncol.

[57] Kuwata T., Yanagihara K., Iino Y. (2019). Establishment of novel gastric cancer patient-derived xenografts and cell lines: pathological comparison between primary tumor, patient-derived, and cell-line derived xenografts. Cells.

[58] Heo E.J., Cho Y.J., Cho W.C. (2017). Patient-derived xenograft models of epithelial ovarian cancer for preclinical studies. Cancer Res Treat.

[59] Centenera M.M., Hickey T.E., Jindal S. (2018). A patient-derived explant (PDE) model of hormone-dependent cancer. Mol Oncol.

[60] Cho S.Y., Kang W., Han J.Y. (2016). An integrative approach to precision cancer medicine using patient-derived xenografts. Mol Cell.

[61] Lin D., Wyatt A.W., Xue H. (2014). High fidelity patient-derived xenografts for accelerating prostate cancer discovery and drug development. Cancer Res.

[62] Wu P., Xu R., Chen X. (2020). Establishment and characterization of patient-derived xenografts for hormone-naïve and castrate-resistant prostate cancers to improve treatment modality evaluation. Aging.

[63] Liu B., Zhou M., Li X. (2021). Interrogation of gender disparity uncovers androgen receptor as the transcriptional activator for oncogenic miR-125b in gastric cancer. Cell Death Dis.

[64] Curtis C., Shah S.P., Chin S.F. (2012). The genomic and transcriptomic architecture of 2000 breast tumours reveals novel subgroups. Nature.

[65] Stripecke R., Münz C., Schuringa J.J. (2020). Innovations, challenges, and minimal information for standardization of humanized mice. EMBO Mol Med.

[66] Olson B., Li Y., Lin Y., Liu E.T., Patnaik A. (2018). Mouse models for cancer immunotherapy research. Cancer Discov.

[67] Johnson J.I., Decker S., Zaharevitz D. (2001). Relationships between drug activity in NCI preclinical *in vitro* and *in vivo* models and early clinical trials. Br J Cancer.

[68] Mer A.S., Ba-Alawi W., Smirnov P. (2019). Integrative pharmacogenomics analysis of patient-derived xenografts. Cancer Res.

[69] Villafranca-Magdalena B., Masferrer-Ferragutcasas C., Lopez-Gil C. (2022). Genomic validation of endometrial cancer patient-derived xenograft models as a preclinical tool. Int J Mol Sci.

[70] DeCarvalho A.C., Kim H., Poisson L.M. (2018). Discordant inheritance of chromosomal and extrachromosomal DNA elements contributes to dynamic disease evolution in glioblastoma. Nat Genet.

[71] Julien S., Merino-Trigo A., Lacroix L. (2012). Characterization of a large panel of patient-derived tumor xenografts representing the clinical heterogeneity of human colorectal cancer. Clin Cancer Res.

[72] DeRose Y.S., Wang G., Lin Y.C. (2011). Tumor grafts derived from women with breast cancer authentically reflect tumor pathology, growth, metastasis and disease outcomes. Nat Med.

[73] Braekeveldt N., von Stedingk K., Fransson S. (2018). Patient-derived xenograft models reveal intratumor heterogeneity and temporal stability in neuroblastoma. Cancer Res.

[74] Hodgson J.G., Yeh R.F., Ray A. (2009). Comparative analyses of gene copy number and mRNA expression in glioblastoma multiforme tumors and xenografts. Neuro Oncol.

[75] Kuijjer M.L., Namløs H.M., Hauben E.I. (2011). mRNA expression profiles of primary high-grade central osteosarcoma are preserved in cell lines and xenografts. BMC Med Genom.

[76] Bogner P.N., Patnaik S.K., Pitoniak R. (2009). Lung cancer xenografting alters microRNA profile but not immunophenotype. Biochem Biophys Res Commun.

[77] Yao Y.M.M., Donoho G.P., Iversen P.W. (2017). Mouse PDX trial suggests synergy of concurrent inhibition of RAF and EGFR in colorectal cancer with *BRAF* or *KRAS* mutations. Clin Cancer Res.

[78] Nunes M., Vrignaud P., Vacher S. (2015). Evaluating patient-derived colorectal cancer xenografts as preclinical models by comparison with patient clinical data. Cancer Res.

[79] Siolas D., Hannon G.J. (2013). Patient-derived tumor xenografts: transforming clinical samples into mouse models. Cancer Res.

[80] Hauge A., Wegner C.S., Gaustad J.V., Simonsen T.G., Andersen L.M.K., Rofstad E.K. (2017). DCE-MRI of patient-derived xenograft models of uterine cervix carcinoma: associations with parameters of the tumor microenvironment. J Transl Med.

[81] Haldorsen I.S., Popa M., Fonnes T. (2015). Multimodal imaging of orthotopic mouse model of endometrial carcinoma. PLoS One.

[82] Moss J.I., Barjat H., Emmas S.A. (2020). High-resolution 3D visualization of nanomedicine distribution in tumors. Theranostics.

[83] Guo W., Giancotti F.G. (2004). Integrin signalling during tumour progression. Nat Rev Mol Cell Biol.

[84] Hynes R.O. (1992). Integrins: versatility, modulation, and signaling in cell adhesion. Cell.

[85] Sprouffske K., Kerr G., Li C. (2020). Genetic heterogeneity and clonal evolution during metastasis in breast cancer patient-derived tumor xenograft models. Comput Struct Biotechnol J.

[86] Fidler I.J. (2003). The pathogenesis of cancer metastasis: the ‘seed and soil’ hypothesis revisited. Nat Rev Cancer.

[87] Blomme A., Van Simaeys G., Doumont G. (2018). Murine stroma adopts a human-like metabolic phenotype in the PDX model of colorectal cancer and liver metastases. Oncogene.

[88] Naseer A., Terry A., Gilroy K. (2015). Frequent infection of human cancer xenografts with murine endogenous retroviruses *in vivo*. Viruses.

[89] Yuan Z., Fan X., Zhu J.J. (2021). Presence of complete murine viral genome sequences in patient-derived xenografts. Nat Commun.

[90] Bock S., Mullins C.S., Klar E., Pérot P., Maletzki C., Linnebacher M. (2018). Murine endogenous retroviruses are detectable in patient-derived xenografts but not in patient-individual cell lines of human colorectal cancer. Front Microbiol.

[91] Choi Y.Y., Lee J.E., Kim H. (2016). Establishment and characterisation of patient-derived xenografts as paraclinical models for gastric cancer. Sci Rep.

[92] Gendoo D.M.A. (2020). Bioinformatics and computational approaches for analyzing patient-derived disease models in cancer research. Comput Struct Biotechnol J.

[93] Meehan T.F., Conte N., Goldstein T. (2017). PDX-MI: minimal information for patient-derived tumor xenograft models. Cancer Res.

[94] Landuzzi L., Manara M.C., Lollini P.L., Scotlandi K. (2021). Patient derived xenografts for genome-driven therapy of osteosarcoma. Cells.

[95] Borodovsky A., McQuiston T.J., Stetson D. (2017). Generation of stable PDX derived cell lines using conditional reprogramming. Mol Cancer.

[96] Nanni P., Landuzzi L., Manara M.C. (2019). Bone sarcoma patient-derived xenografts are faithful and stable preclinical models for molecular and therapeutic investigations. Sci Rep.

[97] Pham K., Delitto D., Knowlton A.E. (2016). Isolation of pancreatic cancer cells from a patient-derived xenograft model allows for practical expansion and preserved heterogeneity in culture. Am J Pathol.

[98] Vaeteewoottacharn K., Pairojkul C., Kariya R. (2019). Establishment of highly transplantable cholangiocarcinoma cell lines from a patient-derived xenograft mouse model. Cells.

[99] Long Y., Xie B., Shen H.C., Wen D. (2022). Translation potential and challenges of *in vitro* and murine models in cancer clinic. Cells.

[100] Frank N.D., Jones M.E., Vang B., Coeshott C. (2019). Evaluation of reagents used to coat the hollow-fiber bioreactor membrane of the Quantum® Cell Expansion System for the culture of human mesenchymal stem cells. Mater Sci Eng C Mater Biol Appl.

[101] Li Z., Zheng W., Wang H. (2021). Application of animal models in cancer research: recent progress and future prospects. Cancer Manag Res.

[102] Zhang F., Wang W., Long Y. (2018). Characterization of drug responses of mini patient-derived xenografts in mice for predicting cancer patient clinical therapeutic response. Cancer Commun.

[103] Qu J., Kalyani F.S., Liu L., Cheng T., Chen L. (2021). Tumor organoids: synergistic applications, current challenges, and future prospects in cancer therapy. Cancer Commun.

[104] Kopper O., de Witte C.J., Lõhmussaar K. (2019). An organoid platform for ovarian cancer captures intra- and interpatient heterogeneity. Nat Med.

[105] Xu H., Lyu X., Yi M., Zhao W., Song Y., Wu K. (2018). Organoid technology and applications in cancer research. J Hematol Oncol.

[106] Vlachogiannis G., Hedayat S., Vatsiou A. (2018). Patient-derived organoids model treatment response of metastatic gastrointestinal cancers. Science.

[107] Xu X., Kumari R., Zhou J. (2023). A living biobank of matched pairs of patient-derived xenografts and organoids for cancer pharmacology. PLoS One.

[108] Wilde B.R., Kaadige M.R., Guillen K.P., Butterfield A., Welm B.E., Ayer D.E. (2020). Protein synthesis inhibitors stimulate MondoA transcriptional activity by driving an accumulation of glucose 6-phosphate. Cancer Metabol.

[109] Aizawa Y., Takada K., Aoyama J. (2023). Establishment of experimental salivary gland cancer models using organoid culture and patient-derived xenografting. Cell Oncol.

[110] Wang Z., He X., Qiao H., Chen P. (2020). Global trends of organoid and organ-on-a-chip in the past decade: a bibliometric and comparative study. Tissue Eng Part A.

[111] Xu H., Jiao D., Liu A., Wu K. (2022). Tumor organoids: applications in cancer modeling and potentials in precision medicine. J Hematol Oncol.

[112] Shultz L.D., Brehm M.A., Garcia-Martinez J.V., Greiner D.L. (2012). Humanized mice for immune system investigation: progress, promise and challenges. Nat Rev Immunol.

[113] Martinov T., McKenna K.M., Tan W.H. (2021). Building the next generation of humanized hemato-lymphoid system mice. Front Immunol.

[114] Zitvogel L., Pitt J.M., Daillère R., Smyth M.J., Kroemer G. (2016). Mouse models in oncoimmunology. Nat Rev Cancer.

[115] Melkus M.W., Estes J.D., Padgett-Thomas A. (2006). Humanized mice mount specific adaptive and innate immune responses to EBV and TSST-1. Nat Med.

[116] Rongvaux A., Willinger T., Martinek J. (2014). Development and function of human innate immune cells in a humanized mouse model. Nat Biotechnol.

[117] Kersten K., de Visser K.E., van Miltenburg M.H., Jonkers J. (2017). Genetically engineered mouse models in oncology research and cancer medicine. EMBO Mol Med.

[118] Peng W., Chen J.Q., Liu C. (2016). Loss of PTEN promotes resistance to T cell-mediated immunotherapy. Cancer Discov.

[119] Schönhuber N., Seidler B., Schuck K. (2014). A next-generation dual-recombinase system for time- and host-specific targeting of pancreatic cancer. Nat Med.

[120] Maresch R., Mueller S., Veltkamp C. (2016). Multiplexed pancreatic genome engineering and cancer induction by transfection-based CRISPR/Cas9 delivery in mice. Nat Commun.

[121] McFadden D.G., Politi K., Bhutkar A. (2016). Mutational landscape of EGFR-, MYC-, and Kras-driven genetically engineered mouse models of lung adenocarcinoma. Proc Natl Acad Sci U S A.

[122] Ku S.Y., Rosario S., Wang Y. (2017). Rb1 and Trp53 cooperate to suppress prostate cancer lineage plasticity, metastasis, and antiandrogen resistance. Science.

[123] Lee Y., Grill S., Sanchez A., Murphy-Ryan M., Poss K.D. (2005). Fgf signaling instructs position-dependent growth rate during zebrafish fin regeneration. Development.

[124] Costa B., Estrada M.F., Mendes R.V., Fior R. (2020). Zebrafish avatars towards personalized medicine - a comparative review between avatar models. Cells.

[125] Kirchberger S., Sturtzel C., Pascoal S., Distel M. (2017). *Quo natas, danio? -* recent progress in modeling cancer in zebrafish. Front Oncol.

[126] Howe K., Clark M.D., Torroja C.F. (2013). The zebrafish reference genome sequence and its relationship to the human genome. Nature.

[127] Fior R., Póvoa V., Mendes R.V. (2017). Single-cell functional and chemosensitive profiling of combinatorial colorectal therapy in zebrafish xenografts. Proc Natl Acad Sci U S A.

[128] Letrado P., de Miguel I., Lamberto I., Díez-Martínez R., Oyarzabal J. (2018). Zebrafish: speeding up the cancer drug discovery process. Cancer Res.

[129] Goldstone J.V., McArthur A.G., Kubota A. (2010). Identification and developmental expression of the full complement of cytochrome P450 genes in zebrafish. BMC Genom.

[130] Astone M., Dankert E.N., Alam S.K., Hoeppner L.H. (2017). Fishing for cures: the alLURE of using zebrafish to develop precision oncology therapies. npj Precis Oncol.

[131] Bangi E., Ang C., Smibert P. (2019). A personalized platform identifies trametinib plus zoledronate for a patient with KRAS-mutant metastatic colorectal cancer. Sci Adv.

[132] Pandey U.B., Nichols C.D. (2011). Human disease models in *Drosophila melanogaster* and the role of the fly in therapeutic drug discovery. Pharmacol Rev.

[133] Gonzalez C. (2013). *Drosophila melanogaster*: a model and a tool to investigate malignancy and identify new therapeutics. Nat Rev Cancer.

[134] Kamdem J.P., Duarte A.E., Ibrahim M., Lukong K.E., Barros L.M., Roeder T. (2020). Bibliometric analysis of personalized humanized mouse and *Drosophila* models for effective combinational therapy in cancer patients. Biochim Biophys Acta, Mol Basis Dis.

[135] Chu P.Y., Koh A.P.F., Antony J., Huang R.Y.J. (2022). Applications of the chick chorioallantoic membrane as an alternative model for cancer studies. Cells Tissues Organs.

[136] Idrisova K.F., Simon H.U., Gomzikova M.O. (2022). Role of patient-derived models of cancer in translational oncology. Cancers.

[137] Gardner H.L., Fenger J.M., London C.A. (2016). Dogs as a model for cancer. Annu Rev Anim Biosci.

[138] Ressel L., Millanta F., Caleri E., Innocenti V.M., Poli A. (2009). Reduced PTEN protein expression and its prognostic implications in canine and feline mammary tumors. Vet Pathol.

[139] Wang K., Jin Q., Ruan D. (2017). Cre-dependent Cas9-expressing pigs enable efficient *in vivo* genome editing. Genome Res.

[140] Jin Q., Liu X., Zhuang Z. (2023). Doxycycline-dependent Cas9-expressing pig resources for conditional *in vivo* gene nullification and activation. Genome Biol.

[141] Tu Q., Yang D., Zhang X. (2019). A novel pancreatic cancer model originated from transformation of acinar cells in adult tree shrew, a primate-like animal. Dis Model Mech.

[142] Ledford H. (2016). US cancer institute to overhaul tumour cell lines. Nature.

[143] Bray F., Ferlay J., Soerjomataram I., Siegel R.L., Torre L.A., Jemal A. (2018). Global cancer statistics 2018: GLOBOCAN estimates of incidence and mortality worldwide for 36 cancers in 185 countries. CA Cancer J Clin.

[144] Grunblatt E., Wu N., Zhang H. (2020). *MYCN* drives chemoresistance in small cell lung cancer while USP7 inhibition can restore chemosensitivity. Genes Dev.

[145] Ali Z., Vildevall M., Rodriguez G.V. (2022). Zebrafish patient-derived xenograft models predict lymph node involvement and treatment outcome in non-small cell lung cancer. J Exp Clin Cancer Res.

[146] Arnold M., Abnet C.C., Neale R.E. (2020). Global burden of 5 major types of gastrointestinal cancer. Gastroenterology.

[147] Liu X., Meltzer S.J. (2017). Gastric cancer in the era of precision medicine. Cell Mol Gastroenterol Hepatol.

[148] Yang Y.M., Hong P., Xu W.W., He Q.Y., Li B. (2020). Advances in targeted therapy for esophageal cancer. Signal Transduct Targeted Ther.

[149] Sun F.X., Tang Z.Y., Lui K.D. (1996). Establishment of a metastatic model of human hepatocellular carcinoma in nude mice via orthotopic implantation of histologically intact tissues. Int J Cancer.

[150] Tan X.P., He Y., Yang J. (2023). Blockade of NMT1 enzymatic activity inhibits N-myristoylation of VILIP3 protein and suppresses liver cancer progression. Signal Transduct Targeted Ther.

[151] Huang C.S., Zhu Y.Q., Xu Q.C. (2022). YTHDF2 promotes intrahepatic cholangiocarcinoma progression and desensitises cisplatin treatment by increasing CDKN1B mRNA degradation. Clin Transl Med.

[152] Bissig-Choisat B., Kettlun-Leyton C., Legras X.D. (2016). Novel patient-derived xenograft and cell line models for therapeutic testing of pediatric liver cancer. J Hepatol.

[153] Ma J., Xu X., Fu C. (2022). CDH17 nanobodies facilitate rapid imaging of gastric cancer and efficient delivery of immunotoxin. Biomater Res.

[154] Akagi S., Ando H., Fujita K. (2021). Therapeutic efficacy of a paclitaxel-loaded nanofibrillated bacterial cellulose (PTX/NFBC) formulation in a peritoneally disseminated gastric cancer xenograft model. Int J Biol Macromol.

[155] Tang L., Mei L.J., Yang X.J. (2011). Cytoreductive surgery plus hyperthermic intraperitoneal chemotherapy improves survival of gastric cancer with peritoneal carcinomatosis: evidence from an experimental study. J Transl Med.

[156] Zheng L., Liang H., Zhang Q. (2022). circPTEN1, a circular RNA generated from PTEN, suppresses cancer progression through inhibition of TGF-β/Smad signaling. Mol Cancer.

[157] Zeng K., Peng J., Xing Y. (2023). A positive feedback circuit driven by m^6^A-modified circular RNA facilitates colorectal cancer liver metastasis. Mol Cancer.

[158] Li W., Zhou C., Yu L. (2024). Tumor-derived lactate promotes resistance to bevacizumab treatment by facilitating autophagy enhancer protein RUBCNL expression through histone H3 lysine 18 lactylation (H3K18la) in colorectal cancer. Autophagy.

[159] Li C., Zhang K., Pan G. (2021). Dehydrodiisoeugenol inhibits colorectal cancer growth by endoplasmic reticulum stress-induced autophagic pathways. J Exp Clin Cancer Res.

[160] Kendzia S., Franke S., Kröhler T. (2023). A combined computational and functional approach identifies IGF_2_BP_2_ as a driver of chemoresistance in a wide array of pre-clinical models of colorectal cancer. Mol Cancer.

[161] Chen S., Zhang L., Li M. (2022). *Fusobacterium nucleatum* reduces METTL3-mediated m^6^A modification and contributes to colorectal cancer metastasis. Nat Commun.

[162] Chu J., Niu X., Chang J. (2020). Metabolic remodeling by TIGAR overexpression is a therapeutic target in esophageal squamous-cell carcinoma. Theranostics.

[163] Mizumoto A., Ohashi S., Kamada M. (2019). Combination treatment with highly bioavailable curcumin and NQO1 inhibitor exhibits potent antitumor effects on esophageal squamous cell carcinoma. J Gastroenterol.

[164] Sung H., Ferlay J., Siegel R.L. (2021). Global cancer statistics 2020: GLOBOCAN estimates of incidence and mortality worldwide for 36 cancers in 185 countries. CA Cancer J Clin.

[165] Stossel C., Raitses-Gurevich M., Atias D. (2023). Spectrum of response to platinum and PARP inhibitors in germline BRCA-associated pancreatic cancer in the clinical and preclinical setting. Cancer Discov.

[166] Abdolahi S., Ghazvinian Z., Muhammadnejad S., Saleh M., Asadzadeh Aghdaei H., Baghaei K. (2022). Patient-derived xenograft (PDX) models, applications and challenges in cancer research. J Transl Med.

[167] Guillen K.P., Fujita M., Butterfield A.J. (2022). A human breast cancer-derived xenograft and organoid platform for drug discovery and precision oncology. Nat Cancer.

[168] Navarro-Yepes J., Kettner N.M., Rao X. (2023). Abemaciclib is effective in palbociclib-resistant hormone receptor-positive metastatic breast cancers. Cancer Res.

[169] Kurani H., Razavipour S.F., Harikumar K.B. (2022). DOT1L is a novel cancer stem cell target for triple-negative breast cancer. Clin Cancer Res.

[170] Sa J.K., Hwang J.R., Cho Y.J. (2019). Pharmacogenomic analysis of patient-derived tumor cells in gynecologic cancers. Genome Biol.

[171] Chowdhury S., Kennedy J.J., Ivey R.G. (2023). Proteogenomic analysis of chemo-refractory high-grade serous ovarian cancer. Cell.

[172] Beaver J.A., Coleman R.L., Arend R.C. (2019). Advancing drug development in gynecologic malignancies. Clin Cancer Res.

[173] Van Nyen T., Planque M., van Wagensveld L. (2022). Serine metabolism remodeling after platinum-based chemotherapy identifies vulnerabilities in a subgroup of resistant ovarian cancers. Nat Commun.

[174] Zhou J., Gelot C., Pantelidou C. (2021). A first-in-class polymerase *Theta* inhibitor selectively targets homologous-recombination-deficient tumors. Nat Cancer.

[175] Zhang X., Yao J., Li X. (2023). Targeting polyploid giant cancer cells potentiates a therapeutic response and overcomes resistance to PARP inhibitors in ovarian cancer. Sci Adv.

[176] Ghilardi C., Moreira-Barbosa C., Brunelli L. (2022). PGC1α/β expression predicts therapeutic response to oxidative phosphorylation inhibition in ovarian cancer. Cancer Res.

[177] Tanaka T., Nishie R., Ueda S. (2021). Patient-derived xenograft models in cervical cancer: a systematic review. Int J Mol Sci.

[178] Liu L., Wu M., Huang A. (2023). Establishment of a high-fidelity patient-derived xenograft model for cervical cancer enables the evaluation of patient’s response to conventional and novel therapies. J Transl Med.

[179] Trubicka J., Grajkowska W., Dembowska-Bagińska B. (2022). Molecular markers of pediatric solid tumors-diagnosis, optimizing treatments, and determining susceptibility: current state and future directions. Cells.

[180] Kushner B.H. (2004). Neuroblastoma: a disease requiring a multitude of imaging studies. J Nucl Med.

[181] Tsuchida Y., Yokomori K., Iwanaka T., Saito S. (1984). Nude mouse xenograft study for treatment of neuroblastoma: effects of chemotherapeutic agents and surgery on tumor growth and cell kinetics. J Pediatr Surg.

[182] Matthay K.K., Maris J.M., Schleiermacher G. (2016). Neuroblastoma. Nat Rev Dis Prim.

[183] Pugh T.J., Morozova O., Attiyeh E.F. (2013). The genetic landscape of high-risk neuroblastoma. Nat Genet.

[184] Kamili A., Atkinson C., Trahair T.N., Fletcher J.I. (2020). Mouse models of high-risk neuroblastoma. Cancer Metastasis Rev.

[185] Tucker E.R., Jiménez I., Chen L. (2023). Combination therapies targeting ALK-aberrant neuroblastoma in preclinical models. Clin Cancer Res.

[186] Pascual-Pasto G., Castillo-Ecija H., Unceta N. (2022). SPARC-mediated long-term retention of nab-paclitaxel in pediatric sarcomas. J Contr Release.

[187] Chen D., Lei C., Liu W. (2023). Reduction-responsive nucleic acid nanocarrier-mediated miR-22 inhibition of PI3K/AKT pathway for the treatment of patient-derived tumor xenograft osteosarcoma. Bioact Mater.

[188] Schott C.R., Koehne A.L., Sayles L.C. (2024). Osteosarcoma PDX-derived cell line models for preclinical drug evaluation demonstrate metastasis inhibition by dinaciclib through a genome-targeted approach. Clin Cancer Res.

[189] Sobel R.E., Sadar M.D. (2005). Cell lines used in prostate cancer research: a compendium of old and new lines: Part 1. J Urol.

[190] Namekawa T., Ikeda K., Horie-Inoue K., Inoue S. (2019). Application of prostate cancer models for preclinical study: advantages and limitations of cell lines, patient-derived xenografts, and three-dimensional culture of patient-derived cells. Cells.

[191] Palanisamy N., Yang J., Shepherd P.D.A. (2020). The MD Anderson prostate cancer patient-derived xenograft series (MDA PCa PDX) captures the molecular landscape of prostate cancer and facilitates marker-driven therapy development. Clin Cancer Res.

[192] Wen S., Wei Y., Zen C., Xiong W., Niu Y., Zhao Y. (2020). Long non-coding RNA NEAT1 promotes bone metastasis of prostate cancer through N6-methyladenosine. Mol Cancer.

[193] Guo C., Figueiredo I., Gurel B. (2023). B7-H3 as a therapeutic target in advanced prostate cancer. Eur Urol.

[194] Li J., Huang T., Hua J. (2023). CD46 targeted ^212^Pb alpha particle radioimmunotherapy for prostate cancer treatment. J Exp Clin Cancer Res.

[195] Zhang H., Xiao X., Wei W. (2021). CircLIFR synergizes with MSH2 to attenuate chemoresistance via MutSα/ATM-p73 axis in bladder cancer. Mol Cancer.

[196] Tatum J.L., Kalen J.D., Jacobs P.M. (2022). 3'-[^18^F]fluoro-3'-deoxythymidine ([^18^F]FLT) positron emission tomography as an *in vivo* biomarker of inhibition of CDK 4/6-Rb pathway by palbociclib in a patient derived bladder tumor. J Transl Med.

[197] An M., Zheng H., Huang J. (2022). Aberrant nuclear export of circNCOR1 underlies SMAD7-mediated lymph node metastasis of bladder cancer. Cancer Res.

[198] Huang M., Dong W., Xie R. (2022). HSF_1_ facilitates the multistep process of lymphatic metastasis in bladder cancer via a novel PRMT5-WDR5-dependent transcriptional program. Cancer Commun.

[199] Jin N., Keam B., Cho J. (2021). Therapeutic implications of activating noncanonical PIK3CA mutations in head and neck squamous cell carcinoma. J Clin Invest.

[200] Pohl L., Friedhoff J., Jurcic C. (2022). Kidney cancer models for pre-clinical drug discovery: challenges and opportunities. Front Oncol.

[201] Wei S., Yin D., Yu S. (2022). Antitumor activity of a mitochondrial-targeted HSP90 inhibitor in gliomas. Clin Cancer Res.

[202] Haikala H.M., Lopez T., Köhler J. (2022). EGFR inhibition enhances the cellular uptake and antitumor-activity of the HER3 antibody-drug conjugate HER3-DXd. Cancer Res.

[203] Majeed S., Aparnathi M.K., Nixon K.C.J. (2022). Targeting the ubiquitin-proteasome system using the UBA1 inhibitor TAK-243 is a potential therapeutic strategy for small-cell lung cancer. Clin Cancer Res.

[204] Cao Y., Wang J., Tian H., Fu G.H. (2020). Mitochondrial ROS accumulation inhibiting JAK2/STAT3 pathway is a critical modulator of CYT997-induced autophagy and apoptosis in gastric cancer. J Exp Clin Cancer Res.

[205] Liu C., Shen A., Song J. (2024). LncRNA-CCAT5-mediated crosstalk between Wnt/β-catenin and STAT3 signaling suggests novel therapeutic approaches for metastatic gastric cancer with high Wnt activity. Cancer Commun.

[206] Yin F., Zhao R., Gorja D.R. (2022). Novel dual inhibitor for targeting PIM1 and FGFR1 kinases inhibits colorectal cancer growth *in vitro* and patient-derived xenografts *in vivo*. Acta Pharm Sin B.

[207] Tang C., Ke M., Yu X. (2023). GART functions as a novel methyltransferase in the RUVBL1/β-catenin signaling pathway to promote tumor stemness in colorectal cancer. Adv Sci.

[208] Jia X., Wang P., Huang C. (2023). Toosendanin targeting eEF2 impedes topoisomerase I & II protein translation to suppress esophageal squamous cell carcinoma growth. J Exp Clin Cancer Res.

[209] Johnston M.E., Rivas M.P., Nicolle D. (2021). Olaparib inhibits tumor growth of hepatoblastoma in patient-derived xenograft models. Hepatology.

[210] Ruan Y., Chen T., Zheng L. (2023). cDCBLD2 mediates sorafenib resistance in hepatocellular carcinoma by sponging miR-345-5p binding to the TOP2A coding sequence. Int J Biol Sci.

[211] Liu W., Yu X., Yuan Y. (2023). CD73, a promising therapeutic target of diclofenac, promotes metastasis of pancreatic cancer through a nucleotidase independent mechanism. Adv Sci.

[212] Chew N.J., Lim Kam Sian T.C.C., Nguyen E.V. (2021). Evaluation of FGFR targeting in breast cancer through interrogation of patient-derived models. Breast Cancer Res.

[213] Ciscar M., Trinidad E.M., Perez-Chacon G. (2023). RANK is a poor prognosis marker and a therapeutic target in ER-negative postmenopausal breast cancer. EMBO Mol Med.

[214] Hossen M.N., Wang L., Dwivedi S.K.D. (2022). Gold nanoparticles disrupt the IGFBP2/mTOR/PTEN axis to inhibit ovarian cancer growth. Adv Sci.

[215] Xiao R., You L., Zhang L. (2022). Inhibiting the IRE1α axis of the unfolded protein response enhances the antitumor effect of AZD1775 in TP53 mutant ovarian cancer. Adv Sci.

[216] Ye M., Liu T., Miao L. (2023). The role of ZNF275/AKT pathway in carcinogenesis and cisplatin chemosensitivity of cervical cancer using patient-derived xenograft models. Cancers.

[217] Hansson K., Radke K., Aaltonen K. (2020). Therapeutic targeting of KSP in preclinical models of high-risk neuroblastoma. Sci Transl Med.

[218] Nomura M., Rainusso N., Lee Y.C. (2019). Tegavivint and the β-catenin/ALDH axis in chemotherapy-resistant and metastatic osteosarcoma. J Natl Cancer Inst.

[219] Liu B., Li L., Yang G. (2019). PARP inhibition suppresses GR-MYCN^-^CDK5-RB1-E2F1 signaling and neuroendocrine differentiation in castration-resistant prostate cancer. Clin Cancer Res.

[220] Steele T.M., Tsamouri M.M., Siddiqui S. (2023). Cisplatin-induced increase in heregulin 1 and its attenuation by the monoclonal ErbB3 antibody seribantumab in bladder cancer. Sci Rep.

[221] Wu Y., Chen S., Yang X. (2023). Combining the tyrosine kinase inhibitor cabozantinib and the mTORC1/2 inhibitor sapanisertib blocks ERK pathway activity and suppresses tumor growth in renal cell carcinoma. Cancer Res.

[222] Yao Y., Wang Y., Chen L. (2022). Clinical utility of PDX cohorts to reveal biomarkers of intrinsic resistance and clonal architecture changes underlying acquired resistance to cetuximab in HNSCC. Signal Transduct Targeted Ther.

[223] Yang E., Wang L., Jin W. (2022). PTRF/Cavin-1 enhances chemo-resistance and promotes temozolomide efflux through extracellular vesicles in glioblastoma. Theranostics.

[224] Mourragui S.M.C., Loog M., Vis D.J. (2021). Predicting patient response with models trained on cell lines and patient-derived xenografts by nonlinear transfer learning. Proc Natl Acad Sci U S A.

[225] Li Q., Dai W., Liu J., Li Y.X., Li Y.Y. (2019). DRAP: a toolbox for drug response analysis and visualization tailored for preclinical drug testing on patient-derived xenograft models. J Transl Med.

